# The Toxicity Phenomenon and the Related Occurrence in Metal and Metal Oxide Nanoparticles: A Brief Review From the Biomedical Perspective

**DOI:** 10.3389/fbioe.2020.00822

**Published:** 2020-07-17

**Authors:** Shokouh Attarilar, Jinfan Yang, Mahmoud Ebrahimi, Qingge Wang, Jia Liu, Yujin Tang, Junlin Yang

**Affiliations:** ^1^Department of Pediatric Orthopaedics, Xin Hua Hospital Affiliated to Shanghai Jiao Tong University School of Medicine, Shanghai, China; ^2^Department of Spine Surgery, Xin Hua Hospital Affiliated to Shanghai Jiao Tong University School of Medicine, Shanghai, China; ^3^National Engineering Research Center of Light Alloy Net Forming, School of Materials Science and Engineering, Shanghai Jiao Tong University, Shanghai, China; ^4^School of Metallurgical Engineering, Xi’an University of Architecture and Technology, Xi’an, China; ^5^Affiliated Hospital of Youjiang Medical University for Nationalities, Baise, China

**Keywords:** non-cytotoxic materials, nanomaterials, cytotoxicity, nanomedicine, metal oxide nanoparticles, nanotoxicology

## Abstract

Thousands of different nanoparticles (NPs) involve in our daily life with various origins from food, cosmetics, drugs, etc. It is believed that decreasing the size of materials up to nanometer levels can facilitate their unfavorable absorption since they can pass the natural barriers of live tissues and organs even, they can go across the relatively impermeable membranes. The interaction of these NPs with the biological environment disturbs the natural functions of cells and its components and cause health issues. In the lack of the detailed and comprehensive standard protocols about the toxicity of NPs materials, their control, and effects, this review study focuses on the current research literature about the related factors in toxicity of NPs such as size, concentration, etc. with an emphasis on metal and metal oxide nanoparticles. The goal of the study is to highlight their potential hazard and the advancement of green non-cytotoxic nanomaterials with safe threshold dose levels to resolve the toxicity issues. This study supports the NPs design along with minimizing the adverse effects of nanoparticles especially those used in biological treatments.

## Introduction

Nanoparticles (NPs) are defined as materials with two dimensions in the range of 1–100 nm (10^–9^ m), while nanomaterials are determined as materials possessing just one dimension in that range according to ASTM E2456 standard (ASTM, 2012). These NPs can have a variety of shapes with different aspect ratios including nanorods with <10 aspect ratio, spherical, cubical, and other possible shapes. Owing to this nanometric size level, NPs can have versatile size-dependent and special properties such as catalytic, electrochemical, optical, magnetic features as well as increased surface to volume ratios which in turn make them the unique materials for modern applications. Metal oxide nanoparticles are amidst the most widely used NPs in a variety of applications including cosmetics (Waghmode et al., 2019), drug and medicine industry (Klȩbowski et al., 2018), detergents, agricultural systems (Chen, 2018), environment (Kanchi and Ahmed, 2018), antibacterial agents (Mordorski and Friedman, 2017), paints and textiles (Vigneshwaran et al., 2010). Nowadays, some metallic NPs including gold NPs (Au NPs), silver NPs (Ag NPs), and metallic magnetic NPs such as iron-oxide NPs (IONPs) are frequently utilized and improved in order to intensify their functions as diagnostic and remedial agents. Table 1 lists some applications of the common metallic NPs.

**TABLE 1:** Application of some metallic and metal oxide nanoparticles.

Metals	Application of metallic and metal oxide nanoparticles
Titanium dioxide (Ti)	Solar cells, food wraps, medicines, pharmaceuticals, lacquers, construction, medical devices, gas sensing, photocatalyst, agriculture, paint, food, cosmetic, sterilization, antibacterial coatings (Waghmode et al., 2019).
Zinc and Zinc oxide (Zn)	Medical and healthcare goods, sunscreens, packaging, UV-protective materials such as textiles.
Aluminum (Al)	Automobile industry, aircraft, heat shielding coatings, military application, corrosion, fuel additive/propellant.
Gold (Au)	Sensory probes, cellular imaging, electronic conductors, drug delivery, therapeutic agents, organic photovoltaics, catalysis, nanofibers, textiles.
Iron (Fe)	Magnetic imaging, environmental remediation, glass and ceramic industry, memory tape, resonance imaging, plastics, nanowires, coatings, textiles, alloy and catalyst applications.
Silica (Si)	Drug and gene delivery, adsorbents, electronic, sensor, catalysis, remediation of the environment pollutants, additive in rubber and plastic industry, filler, electric and thermal insulators.
Silver (Ag)	Antimicrobial coatings, textiles, batteries, surgery, wound dressings, biomedical devices, photography, electrical devices, dental work, burns treatment.
Copper (Cu)	Biosensors and electrochemical sensors, plastic additives like anti-biotic, anti-microbial, and anti-fungal agent, coatings, textiles, nanocomposite coating, catalyst, lubricants, inks, filler.
Cerium (Ce)	Chemical mechanical polishing/planarization, computer chip, corrosion, solar cells, fuel oxidation catalysis, automotive exhaust treatment (Dhall and Self, 2018).
Manganese and its oxides (Mn)	Molecular meshing, solar cells, batteries, catalysts, optoelectronics, drug delivery ion-sieves, imaging agents, magnetic storage devices, water treatment and purification (Hoseinpour and Ghaemi, 2018; Wang W. et al., 2019).
Nickel (Ni)	Fuel cells, membrane fuel cells, automotive catalytic converters, plastics, nanowires, nanofibers, textiles, coatings, conduction, magnetic properties, catalyst, batteries, printing inks.

Metallic NPs’ design and their modification can be done through versatile surface functionalities so they can be conjoined with antibodies, ligands, and drugs, consequently raise their potential applications in biotechnology, drug and gene delivery, magnetic separation and imaging, besides the favorable characteristics they have a potential to cause harmful effects if they enter to live biological systems and tissues (Yang et al., 2018; Gu et al., 2019; Liu et al., 2019; Wang L. et al., 2019). Unfortunately, there are many ways for unwanted and spontaneous entry of NPs to the body system, whether through the air we breathe or the water we drink, also foods, medicines, clothes, and cosmetics are no exception. The main entry routes can be considered as inhalation through the respiratory tract, by transudation through the skin and by ingestion through the digestive tract (Zoroddu et al., 2014). Therefore, nanomaterials released into the body environment seem to be inevitable and may have some unforeseen harmful effects hence it is of crucial importance to study their toxicity-related issues. This subject becomes of more paramount importance if we know that their nanoscale size facilitates their penetration to different live tissues and enables possible interaction with the same sized organs like cells, proteins, and antibodies also they can accumulate in organs and tissues as a foreign body (Nemmar et al., 2002; Nel et al., 2006). This arises from the high surface area for example in the case of two NPs with the same mass, smaller NPs have a larger specific surface area and thus provide a more available area to cellular interactions with nucleic acids, proteins, fatty acids, and carbohydrates (Huang et al., 2017). Considering the so-called issues, a new branch of research was introduced and entitled “Nanotoxicology” which deals with the nanomaterials toxicity (Pacheco et al., 2007). Unfortunately, there have not been yet any comprehensive and precise standard protocols for cytotoxicity of various materials, however, about the NPs, the concentration, composition, size, charge, and other physicochemical factors are considered for material selection and their possible utilization. Various methods are used in order to estimate the cytotoxicity levels of NPs which is categorized into two main groups of *in vitro* and *in vivo* methods. In this regard, the *in vitro* group includes dye exclusion assays (trypan blue exclusion and erythrosin B dye exclusion assays), colorimetric assays (MTT, WST-1, neutral red uptake and lactate dehydrogenase assays), fluorescence-based assays (Alamar blue and protease-based viability assays), luminometric methods (Adenosine triphosphate based method), cell viability test in real-time (estimation of oxidative stress, ROS level measurement, lipid peroxidation, glutathione estimation), apoptosis based assays (Annexin-V FITC/propidium Iodide and TUNEL assays. For determining the level of genotoxicity of nanoparticles *in vitro*, micronucleus formation, cytokinesis block micronucleus, flow micronucleus, and comet assays can be utilized. *In vivo* characterization of toxicity can be done through quantitation and bio-distribution of NPs from tissues, electron microscopy and detection of NPs accumulation, liquid scintillation counting, and NPs’ quantification by drug loading and release. Also, the whole body imaging-based methods are utilized for estimation of NPs’ toxicity and bio-distribution such as *in vivo* optical imaging, computed tomography, magnetic resonance, and nuclear medicine imaging, for more information about cytotoxicity assessment, the readers can refer to (Shah et al., 2020). The main objective of these nanotoxicology experiments and studies is the comprehensive understanding in relation to the toxicity of quantum size effects, shape, and high surface area to volume ratio of nanomaterials in biological environments. In this regard and considering the generally used metal and metal oxide NPs, this review paper focuses on the nanotoxicology of these materials with special attention to the physical properties of NPs and their effects on toxicity. Also, the involved mechanisms in relation to nanotoxicology will be addressed.

## Mechanisms of Nanotoxicology

**FIGURE 1:**
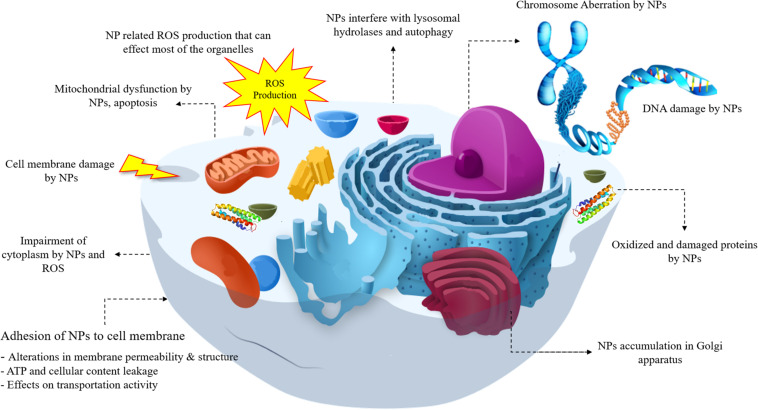
The toxicity mechanisms induced by nanoparticles.

A lot of toxicity mechanism is involved with NPs and the most common types can be listed as below and are shown in Figure 1. As shown in Figure 1, NPs have the ability to interact with most of the cell components from DNA and various proteins to mitochondria, they can lead to reactive oxide species (ROS) formation and affect the different functions of cell. In this regard, DNA damage, lysosomal hydrolases, ROS generation, mitochondrial dysfunction, apoptosis, cell membrane damage, cytoplasm impairment, alterations in ATP, and permeability of cell membrane, accumulation of NPs in Golgi and variations in proteins all can be attributed to NPs interaction.

### Reactive Oxygen Species Formation

The imbalance between production and accumulation of oxygen reactive species (ROS) leads to the occurrence of oxidative stress in cells and live tissues. ROS generates by mitochondria during both physiological and pathological conditions and they are considered as the metabolic by-products of biological systems (Pizzino et al., 2017). They can also be referred to as free radicals and have favorable functions at low or moderate concentrations, they fight with pathogens and are necessary to cell signaling and synthesize various cellular structures and proteins (Dröge, 2002). However, in high concentrations, oxidative stress (OS) condition takes place in which ROS suppress the live cells and organs’ ability to detoxify and unfortunately, it can damage proteins, lipids, and nucleic acids, and severely leads to cell death and disease development including cancer (Katerji et al., 2019).

**FIGURE 2:**
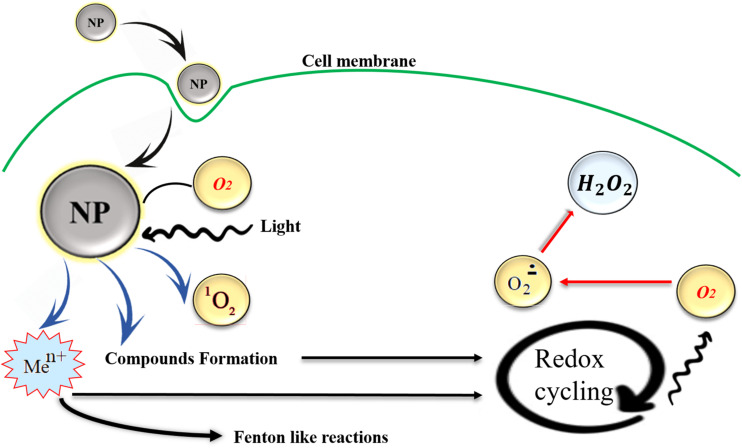
NPs induced ROS production in cells, transition metal ions (Me^*n+*^), or organic compounds may act as initiators of metabolic reactions that generate ROS and they can be released from particle impurities and catalyzing Fenton-type reactions. Also, in some metallic photosensitizer NPs light exposure facilitates the ROS production. Reproduced from Kehrer and Klotz (2015) with permission.

Oxidative stress biomarkers can be categorized in two groups of (a) ROS modified molecules generation and (b) deterioration or derivation of enzymes or antioxidants, the trace of these biomarkers can be detected in body fluids (Tsukahara, 2007). Although due to its unstable condition it is very hard to determine the exact level of ROS, its cellular levels can be measured through various methods such as fluorogenic and fluorescent probes, also hydrogen peroxide (H_2_O_2_), hydroxyl radicals (OH^–^), and peroxyl radicals (ROO^–^) can be estimated by staining methods. In addition, ROS molecules like hydroperoxides (R-OOH) can be quantified by performing the (D-Roms) test through reactive oxygen metabolites derivatives. ROS with a potent chemically reactive characteristic contain oxygen and can be found as superoxides, peroxides, hydroxyl radical, singlet and alpha-oxygen, Figure 2 schematically shows ROS production by NPs, it was believed that some NPs are photosensitizers and they facilitate ROS formation with the light assistance but for the case of tissues which are not exposed to daylight other mechanisms are involved such as organic material released from combustion derived NPs. Also, transition metal ions can be released from particle impurities and catalyzing Fenton-type reactions, for more details about ROS production see Kehrer and Klotz (2015), Saliani et al. (2016), and Flores-López et al. (2019). The ROS produced as one of the natural byproducts of the normal oxygen metabolism and they affect the cell signaling and homeostasis (Devasagayam et al., 2004). In addition to positive functions of ROS formation in cells, their excess generation by external inputs such as NPs can also lead to some harmful effects like apoptosis (programmed cell death) and may induce damages on RNA or DNA (Wan et al., 2012), lipid peroxidation, amino acids oxidation in proteins and deactivation of enzymes by oxidation of co-factors are other unfavorable results of NPs induced ROS generation (Brooker, 2018). The mechanism of ROS production by metallic NPs depends on particle size, shape, surface area, and chemistry. ROS have a key role in multiple cell functions and its biology. ROS generation plays a crucial role in toxicity issues aroused from NPs application, as well as other related phenomena like cellular signaling fluctuations involved in cell death, proliferation, and differentiation (Dayem et al., 2017).

### Cell Damages Through NPs Induced Membrane Perforation

Some metallic NPs like Au NPs can be used in order to maintain unspecific attachment to the cell membrane and activated the interim and cell membrane permeabilization in a spatial manner (Heinemann et al., 2013). Unfortunately, this characteristic can also cause cell damages, for instance Ag NPs with lower than 10 nm diameter have a potential to bind with the cell walls in Escherichia coli bacteria and finally leads to cell death (Gogoi et al., 2006). It was observed (Gopinath et al., 2008) that Ag NPs are able to cause cell apoptosis and damage the mitochondrial membrane during cell apoptosis with cell membrane perforation intervention.

### Cytoskeleton Components Damage

Cytoskeleton acts as a footstone of the cell architecture hence the NPs’ influence on the cytoskeleton network must be carefully considered. Actin and intermediate filaments, microtubules, and different types of proteins are among the most important components of the cytoskeleton (Ispanixtlahuatl-Meráz et al., 2018). Despite the proven non-toxicity of TiO_2_ in most studies (Ding et al., 2016; Zhang et al., 2017) it was reported that TiO_2_ NPs led to actin and tubulin disassembly and some alterations in the cytoskeleton and its proteins (Vuong et al., 2016). TiO_2_ NPs treated epithelial cell line BEAS-2B confirms the expression alterations in mRNAs and miRNAs which is possibly in relation to the cytoskeleton (Thai et al., 2015). The epithelial cells co-culturing and their proteomic analysis indicated that Ag NPs readjust different types of cytokeratins and gelsolin, in contrast to α- and β-tubulin together with actin which were downregulated, and strong dissolution of Ag confirmed the strong effects of NPs rather than Ag ions (Georgantzopoulou et al., 2016). The ZnO NPs can be internalized by endosomes and in turn move to lysosomes, also the existence of zinc ions causes cytotoxicity and actin rearrangement in cell bundles. Besides, this effected tubulin network by ZnO NPs can generate wrapped bundles in the periphery of the nucleus and these improper chromosomes and spindles can subsequently distribute all over the cytoplasm region and cause harmful effects (García-Hevia et al., 2016). Xu F. et al. (2013) reported the cytoskeleton component failure like and filamentous actin (*F*-actin) and the β-tubulin Ag NPs treated samples, it was also demonstrated that they led to the dramatic reduction in the number of synaptic clusters of the presynaptic vesicle protein synaptophysin, and the postsynaptic receptor density protein PSD-95 and lastly Ag NPs cause mitochondria dysfunction in rat cortical cells.

### DNA and Transcription Damage by NPs and Mutagenesis Acceleration

Application of Co NPs within the non-toxic dose range and their exposure to human lung epithelial cell line A549 demonstrated the ROS generation which finally ended to DNA damage. Subsequently, this DNA damage led to ataxia-telangiectasia mutated (ATM) protein activation and increase the phosphorylation of p53 and Rad 51 protein expression, TiO_2_ NPs did not indicate any considerable cytotoxic effects. In addition, Co NPs induced DNA damage is able to actuate various cellular reactions such as apoptosis, cell cycle arrest, and importantly the DNA repair (Wan et al., 2012). The effects of Cu NPs on transcriptional responses of zebrafish embryos confirmed the up-regulation of genes in the healing of wounds and stimulus reactions but it was seen that the genes which are responsible for phototransduction and metabolisms were acted in downward fashion (Zhang et al., 2018). It seems that Cu NPs together with Cu^2+^ ions induce gene transcription damages to Zebrafish embryos (Zhang et al., 2018). The study about the mitotic and meiotic effects of Cu and CdS NPs indicated higher degrees of cytotoxicity in Cu NPs than CdS ones, the mitotic aberrations can be in the result of several phenomena such as (1) DNA depolymerization and sticking of chromosomes bundles, (2) chromosome breakages leading to the generation of rings, bridges, fragments, and micronuclei, (3) prevention of the centromeric division which leads to diplochromosomes formation, (4) spindle apparatus variations which promotes the polyploid cells and laggards. Different mitotic cycles have the potential to initiate the meiotic cell division, NPs inducing aberrations seem to be significant since their consistent changes can cause heritable alterations in the genotype (Kumbhakar et al., 2016).

### Mitochondria Damage

Mitochondria is among the most important organelles of the cells; it chiefly engages in energy supply and differentiation procedure and unfortunately it can be mischievously affected by NPs related toxicity. Mitochondrial permeability transition (PT) occurrence is one of the prime causes of cell death in which a sudden permeability increase in the inner mitochondrial membrane to small size solutes leads to apoptosis, for example, Au NPs with 1.4 nm diameter showed to cause oxidative stress leading to mitochondrial PT in which the higher permeability of mitochondrial membrane toward 1.4 nm Au NPs triggered the cell death by necrosis (Pan et al., 2009). Gallud et al. (2019) also proved the mitochondrial dysfunction in ammonium-modified Au NPs, these cationic Au NPs stimulated autophagy in macrophage-like reporter cells, and cell death can be deteriorated by autophagy inhibition and in general mitochondria-dependent effects of cationic Au NPs induce the quick perish in cells. Yu et al. (2013) reported that ZnO NPs have a capability to affect the mitochondrial membrane potential, also mitochondrial ATP level was significantly diminished in the presence of these ZnO NPs. In addition, interruption of mitochondria, dysfunction, and fall of mitochondrial membrane potential after ZnO NPs treatment to normal skin cells was proven and these NPs adversely influence the mitochondrial network and biogenesis (Yu et al., 2013). Iron-based NPs like Fe_3_O_4_ NPs also can lead to dysfunctions in the mitochondrial activity, increase the ROS production in cells and leads to the draft decrease of ATP level even it can induce autophagy by reduction of cytoplasmic energy (Zhang et al., 2016). These harmful effects are also be seen in TiO_2_ NPs and it was reported that TiO_2_ NPs can cause severe mitochondrial dysfunction, the increment of ROS levels, reduction of ATP generation, mitochondrial phospholipids and metabolic fluxes (Chen et al., 2018). TiO_2_ NPs can also affect the dynamic of the mitochondria and leads to its dynamic imbalances and damages in HT22 Cells and it can also activate the mitochondrial-related apoptosis pathways (Zhao et al., 2019). In the Ag NPs treatment with a diameter of 10 nm it was seen that these NPs are able to impair mitochondrial function and in turn induce cell dysfunctions (Bressan et al., 2013).

### The Effect of NPs on Lysosomes

Lysosomes are defined as membrane-bound organelles comprising hydrolases that act in the deterioration process of macromolecules transported by various pathways including the endocytic, phagocytic, and autophagic ones (Luzio et al., 2014) and they are considered as acute intracellular organelles controlling the cytotoxicity of nanomaterials (Fröhlich, 2013). Metallic NPs like Ag NPs can be taken up by different cell types and they are able to deposite as agglomerates or aggregates in endosomes or lysosomes of the cytoplasm (Guo et al., 2015; Xu et al., 2015). It was shown (Miyayama and Matsuoka, 2016) that Ag NPs exposure on cells can lead to a reduction of Ag dissolution rate (pH-dependent behavior) and MT expression which in turn induce damages on pulmonary epithelial cells. The lysosome impairment was also seen in Fe_3_O_4_ NPs (Zhang et al., 2016), metallic NPs can lead to ROS production and its transportation into lysosomes which finally interfering with the lysosomal hydrolases and induce the autophagy process (Halamoda Kenzaoui et al., 2012). It was seen that TiO_2_ NPs can be responsible for the increment of lysosomal activities mainly caused by oncogenic transformations (Zhu et al., 2012; Lammel et al., 2019). Also, the strength of lysosomal membrane can be significantly decreased by TiO_2_ NPs since they could easily get access into digestive cells, in the next step they can accumulate in lysosomes and then released to the alveolar lumen by apocrine extrusion of residual bodies or by holocrine elimination of dead cells (Jimeno-Romero et al., 2016). The Au NPs can also decrease the lysosomal functions by alkalization of the lysosomal lumen which in turn induce the autophagosomes accumulation and leads to a reduction of cellular degradative capacity and low efficiency in damaged mitochondria release. In fact, these unstable cellular changes absolutely have an influence on the cell functionality, for instance in Au NPs-marked cells, cell migration and invasion were hindered (Manshian et al., 2018).

## Physicochemical Properties of Nanoparticles

**FIGURE 3:**
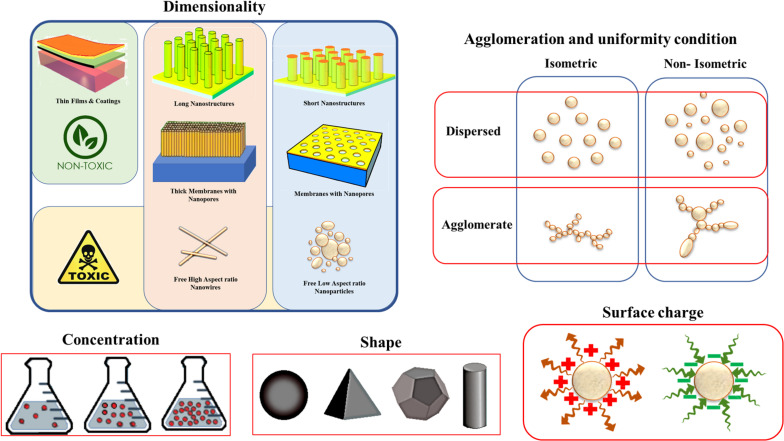
Some physical properties affecting the nanotoxicology of metal-based nanoparticles. Reproduced from Buzea et al. (2007) with permission.

The potency of NPs to enter certain organs across specific pathways and their propensity whether to be accumulated in cell organelles or transported to other organelles is affected by both physical and chemical properties of related NPs. In addition, the physicochemical properties of NPs have a great impact on their toxicity since they can change the mechanism of toxicological response and NPs’ accumulation, uptake, and translocation (Zoroddu et al., 2014). For instance, the same material with different shapes and sizes can considerably change the response of live tissue and identify the destiny of NPs as a safe or toxic one. The important physicochemical properties related to the cytotoxicity of nanomaterials are morphological features like size, shape, roughness and surface area, uniformity of agglomerates and the aggregate formation, mass of NPs, exact chemical composition, concentration or dose of NPs, surface charge, hydrophilicity, solubility and geometrical properties all can influence the behavior of material (Zoroddu et al., 2014). Figure 3 schematically shows some major physical properties related to the toxicity of metal-based NPs in different categories of dimension, agglomerate condition, shape, and size of NPs and the surface charge, also each of which consisted of various states that finally led to toxicity or safety of NPs.

### Size-Dependent Toxicity in Nanoparticles

The NPs’ Size and surface area act as a key factor in its interaction with live tissues, the nanometric size level of NPs are almost in the same range of protein globules ranging from 2 to 10 nm, DNA helix about just two nanometers and cell membrane thickness with 10 nm, so they can easily pass the barriers of cells and enter to cell organelles (Sukhanova et al., 2018). It was shown (De Jong et al., 2008) that the distribution of gold NPs in organs are highly size-dependent, an obvious difference was detected between the distribution of the 10 nm and the larger particles. Ten nm NPs were found in most of the organs whereas the larger NPs distribution was seen in the limited organs of rats. Actually, gold NPs with 6 nm size can freely enter to cell nucleus while these NPs in the size range of 10–16 nm can only be found in cytoplasm and cell membranes which shows the higher toxicity of gold NPs with less than 10 nm size (Huo et al., 2014). Also, it was reported (Pan et al., 2007) that gold NPs with 15 nm size is about sixty times less toxic than 1.4 nm NPs especially for fibroblasts, epithelial cells, macrophages, and melanoma cells. In addition, NPs size can effectively determine and control the interactions between transport and cell defense systems which finally influences the kinetics of NPs distribution and concentration. It is believed that (Zhang S. et al., 2015). NPs with smaller than 5 nm diameter generally can defeat cell entrance barriers and they are able to pass through cell membranes by translocation, while the larger NPs get into the cells by phagocytosis and other possible transportation mechanisms. The *in vivo* experiments (De Jong et al., 2008) confirmed that large NPs can be easily recognized by the immune system and prevents their entrance to the body. The surface area as one of the important factors in NPs cytotoxicity warrants the effective adsorption of NPs on the surface of cell organelles.

### The Effect of Nanoparticles’ Shape on Toxicity

Nanoparticles can have a variety of shapes and geometries including spheres, ellipsoids, cylinders, sheets, cubes, spikes, and rods which considerably affect the toxicity. In this relation, the round-shaped NPs are more susceptible to endocytosis than NPs with fiber and tube geometry (Champion and Mitragotri, 2006). Also, it was indicated that (Zhao et al., 2013) plate-like and needle-like NPs induce larger necrosis proportions than other spherical and rod-like NPs since these shapes have more capacity to induce physical damages to cells and live tissues by direct contact. In addition in gold NPs, geometry and shape of the NPs have an impact on the accumulation kinetics and its excretion and only star-like shapes can be stored in the lung, also it was confirmed that shape and geometrical variations do not considerably increase their chance to pass the blood-brain barriers (Talamini et al., 2017).

### Chemical Composition

Along with other critical factors like shape and size, the chemical composition also must be considered with full attention. Inorganic NPs with the same physical condition but distinct chemical composition confirmed to have different toxicological behaviors. One of the examples is the different toxicity of SiO_2_ and ZnO NPs with 20 nm size in which SiO_2_ induce oxidative stress while ZnO influences the DNA structure (Yang H. et al., 2009). The induced toxicity related to chemical composition mainly arises from metallic ions’ leakage into cells, also some of these metallic NPs are actually has a toxic nature such as As, Pb, Cd, Hg, and Ag since they can damage the cells (Roane et al., 2009). On the other hand, some metals like Fe and Zn are useful from the biological aspect of view but they can be harmful at high concentrations and cause toxicity reactions. Most of the mentioned issues can be solved by coating the NPs cores with polymeric shells, silica layers, or new NPs synthesis methods with non-toxic compounds which can lead to enhanced safety and chemical stability against metal ionic leakages and degradations (Soenen et al., 2015).

### The Effect of Crystal Structure on the Toxicity of NPs

It was shown that the different crystal structures of the same NPs can make alterations in the toxicity response. One of the good examples is TiO_2_ owing to its various crystal structures entitled rutile (TiO_2_ with prism shape), anatase (octahedral crystals), and brookite (orthorhombic crystals). It was reported (Gurr et al., 2005) that 200 nm TiO_2_ NPs with rutile structure caused hydrogen peroxide and oxidative DNA damage, lipid peroxidation, micronuclei formation, and the signs of abnormal chromosome segregation during mitosis process while in the anatase form there was not any considerable toxicity.

### The Effect of Surface Charge on Toxicity

**FIGURE 4:**
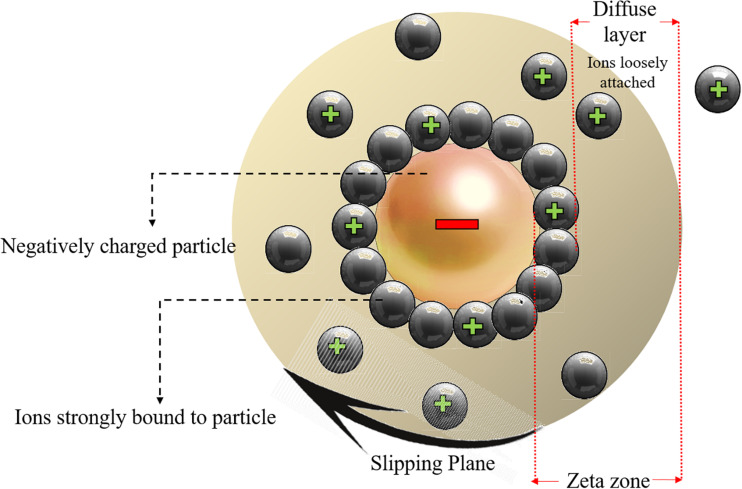
Schematic representation of the zeta potential zone and the electric double layer surrounding the charged nanoparticles. Reproduced from Khan, 2020.

Ionic charges can affect the interaction between the NPs with cells hence having a great impact on toxicity related mechanisms. The surface charge of NPs can be described by zeta potential which is explained as the potential variation among the mobile dispersion medium and the stationary layer of the dispersion medium that is in attachment with the dispersed particle (Lu and Gao, 2010). Figure 4 schematically shows the zeta potential.

The movement of particles in a fluid cause a net surface charge generation which can be defined by zeta potential hence the constancy of particles dispersion can be determined from zeta potential, NPs with a zeta potential value higher than 130 mV or lower than 230 mV are unlikely to aggregate (Khan, 2020), while NPs with lower zeta potential values are prone to stick to each other, entitled as aggregation in which the particles are firmly bonded, or agglomeration if the particles are weakly bonded due to van der Waal’s forces. It was proved that the physical interaction between cellular membrane and NPs is mainly governed by surface charge of NPs and it was also indicated that other toxicity factors like shape and size of NPs have minimal impact on the toxicity of Ag NPs unless the electrostatic barrier between the NPs and cells are overcome. It was shown that positively charged coated Ag NPs are more toxic than that of the negatively charged NPs (El Badawy et al., 2011). Thevenot et al. (2008) demonstrated that negative charged COOH treated TiO_2_ NPs had not an impact on the cell viability because they can easily be absorbed into the cells without any membrane binding. In fact, the positively charged particles are more toxic and the variance surface charge determines the cellular uptake, the positively charged ZnO NPs show increased toxicity values compared to negatively charged NPs (Kim et al., 2014).

### The Effect of NPs Solubility

The metallic NPs that have penetrated the cell is a source for supplying the metal ions and have the potential to constantly release these ions to cytoplasm environment. This metallic ion release is directly dependent on the NPs’ dissolution rate (Khan, 2020). Despite the low dissolution, some metallic ions can show a very toxic behavior in physiological mediums, for example, ZnO NPs with just 10 mg/L dissolved zinc are highly toxic because of its critical concentration and dose range (Khan, 2020) so they should be used in the safe range and the minimum allowed limit must be considered. Horie et al. (2009) reported that NiO NPs have more activity compared to NiO fine particles because the NPs can release higher amounts of Ni^2+^ in the medium while fine particles do not have this capability. In addition, again because of this solubility effect of metallic ions cupric oxide CuO NPs are considerably more toxic than the same amount of CuCl_2_ (Karlsson et al., 2008). It was believed that NPs have higher solubility rate than the bulk materials, this finding can only be correct for NPs in the special size condition (less than 100 nm size) hence particle sizes more than 100 nm fail to enhance the saturation solubility in the low solubility compounds, even if the rate of dissolution is increased, Table 2 shows this situation in more details.

**TABLE 2:** Effect of particle size on the solubility behavior and dissolution rate (He, 2009).

Size of particle	Solubility ratio (S/*S*_*∞*_)*	Variations in dissolution rate
10 μm	1	No considerable effect
1 μm	1.01	10-fold increase
100 nm	1.13	113- fold increase
10 nm	3.32	3320-fold increase

(S/S_∞_)*: the ratio of solid solubility to be dissolved with that of the substance having infinitely large solubility.

## Toxicity of Common Metallic and Metal Oxide NPs

The toxicity phenomenon is a very complicated issue that is dependent on lots of physicochemical parameters, hence different metallic NPs with their special nature would have various toxicity mechanisms and indicate alterations in toxicity amount. It was known that usually as the atomic number of the element increases, cytotoxicity increases (Huang et al., 2017), possibly due to band-gap energy. Also, it was shown that different materials activate certain toxicity mechanisms. In this relation, the present study discusses the involved nanotoxicology mechanisms of common metallic and metal oxide NPs including Ti, Ag, Au, Zn, and Cu, and their effects on biological environments.

### Titanium Dioxide TiO_2_ NPs Toxicity; *in vitro* and *in vivo*

Titanium oxide NPs are among the most manufactured NPs with approximately 10,000 tons yearly production, owing to its unique properties such as suitable strength and Young’s modulus (Ansarian et al., 2019; Attarilar et al., 2020), biocompatibility (Attarilar et al., 2019), corrosion resistance (Gode et al., 2015), solubility properties, surface structure, and the related aggregation manner so it has a lot of applications in industry as listed in Table 1. This wide use of TiO_2_ NPs and its post disposal in the environment may arise the health and ecosystem issues hence its impact on live organisms *in vitro* and *in vivo* must be studied and considered.

**FIGURE 5:**
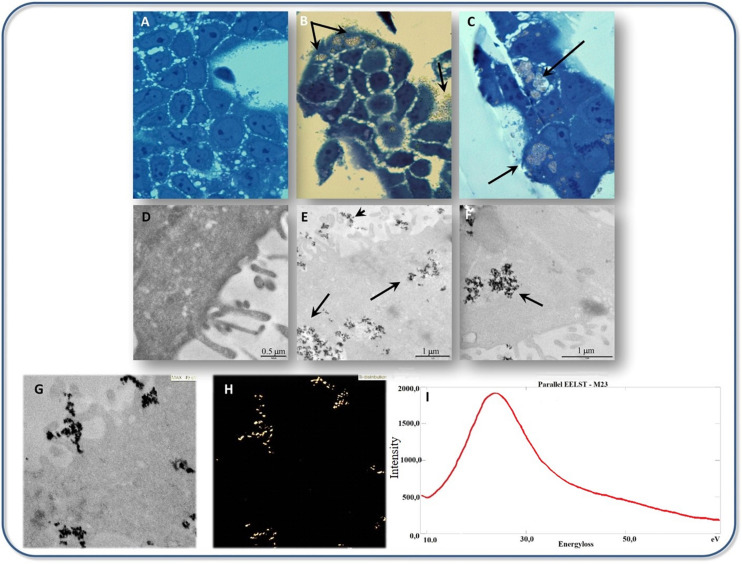
Transmission electron microscope micrographs of TiO_2_ NPs interaction with cultured Caco-2 cells, **(A–C)** Bright field optical microscopy analysis of Caco-2 cells cultured **(A)** without, **(B,C)** with 500 μg/mL TiO_2_ NPs. Black arrows indicate TiO_2_ NPs accumulation observable on the surface of cells as well as in the cytoplasm. **(D–F)** TEM analysis of Caco-2 cells cultured with **(E,F)** or without **(D)** 500 μg/mL TiO_2_ NPs. Black arrows in **(E,F)** indicate TiO_2_ NPs accumulation. **(G,H)** The ultrastructural appearance of Caco-2 cells cultured with 500 μg/mL TiO_2_ NPs **(G)** with the respective map of Ti localization (ESI analysis) is shown in panel **(H)**. **(I)** Electron energy loss spectrum (EELS), withdrawn from the same sample regions of **(G,H)**, is shown. A peak at 25 eV compatible with the TiM_2,3_ edge electron energy loss was detected. Adapted from Pedata et al. (2019) with permission from Elsevier.

The impact of TiO_2_ NPs’ shape on toxicity was examined in BEAS-2B cells, the shape of NPs was selected as bipyramids, rods, and platelets. It was seen that the rod-shaped NPs induced the most amount of toxicity, but in the platelets the genotoxicity and oxidative DNA damage were seen and their accumulation was higher than the rod and bipyramid-shaped NPs (Gea et al., 2019). It seems that among different crystal structures of TiO_2_, the anatase form has more toxicity. De Matteis et al. (2016) indicated that titanium ions are more prone to release in anatase rather than rutile form, also anatase form leads to more ROS production in MCF-7 cell line. Consequently, anatase influences the mitochondrial membrane and is more prone to activate the apoptosis pathway. TiO_2_ NPs treated Caco-2 cell indicated the affected intestinal epithelium layer after 24 h exposure and the cell viability shows the 13% reduction compared to the control sample (Pedata et al., 2019). Transmission electron microscope (TEM) observations showed that TiO_2_ NPs were selectively accumulated in Caco-2 monolayers, as indicated in Figure 5. Also, it was proved that titanium ions have the potential to trigger the production of the pro-inflammatory cytokines and led to some toxic effects on the intestinal epithelium layer (Pedata et al., 2019). Experiments on the A549 cell line (human lung epithelial cells) confirmed the significant cytotoxic effects of citrate-coated TiO_2_ NPs also the DNA damage experiments by comet assay indicated the increasing genotoxic effects in these citrate coated NPs. In fact, variation in the physicochemical properties of NPs by variation in the surface of coating affected the NPs’ toxicity (Stoccoro et al., 2017).

An *in vivo* study about TiO_2_ NPs was done by Fabian et al. (2008), TiO_2_ NPs (<100 nm) were injected to Wistar rats. They did not find any sign of TiO_2_ NPs accumulation in brain and lymph nodes, blood cells, and plasma, the most bioaccumulation of NPs was seen in the liver and lower values of NPs were detected in the kidney, lung, and spleen. TiO_2_ NPs injection into rats at a moderate dose of 20 mg/kg for 20 days had some effects on liver including congestion, prominent vasodilatation, and vacuolization that finally led to liver dysfunction, TiO_2_ NPs injection at high doses (1387 mg/kg body weight) led to mortality of rats after 2 days of injection whereas the low dose injections (in the range of 10 mg/kg body weight) induced toxicity related signs such as decreased water and food consumption, increased number of white blood cells (Ben Younes et al., 2015). Xu J. et al. (2013) showed that TiO_2_ NPs treatment induced some damages in the kidney, lung, brain, spleen, and liver of rats but no considerable pathological effects were detected in rats’ heart. In another study, the rutile TiO_2_ NPs treated rats indicated normal external lung morphology while TiO_2_ NPs in crystalline form with 80% anatase and 20% rutile content showed pulmonary toxicity (Abdelgied et al., 2019). Briefly, it can be said that shape, higher dose, crystalline structure, and phases have the potential to cause toxicity in both *in vitro* and *in vivo* studies hence the careful control of TiO_2_ NPs would be led to more safe utilization of these NPs.

### Silver Nanoparticles and Their Toxicity

Silver as a noble metal in nanoparticle condition is the most widespread antibacterial agent, also Ag salts are utilized as agents for the treatment of different bacterial infections. Consequently, Ag NPs are vastly utilized as bactericides due to its attainment in antibiotic resistance by various bacteria (Sintubin et al., 2012). Ag NPs have the potential to attach to the cell membrane of bacteria or fungi and induce damages on cell membrane structure, intracellular components leakage and in the end cell death (Yamanaka et al., 2005), they can also produce free radicals and cause oxidative stress (Park et al., 2009). Ag NPs are able to construct destructive binding with genomic DNA and prevent the direct replication (Yang W. et al., 2009), it can also decrease the activity of enzymes and other proteins in the transcription stage (Yamanaka et al., 2005), Figure 6 shows Ag NPs interaction with bacterial cells (Marambio-Jones and Hoek, 2010).

**FIGURE 6:**
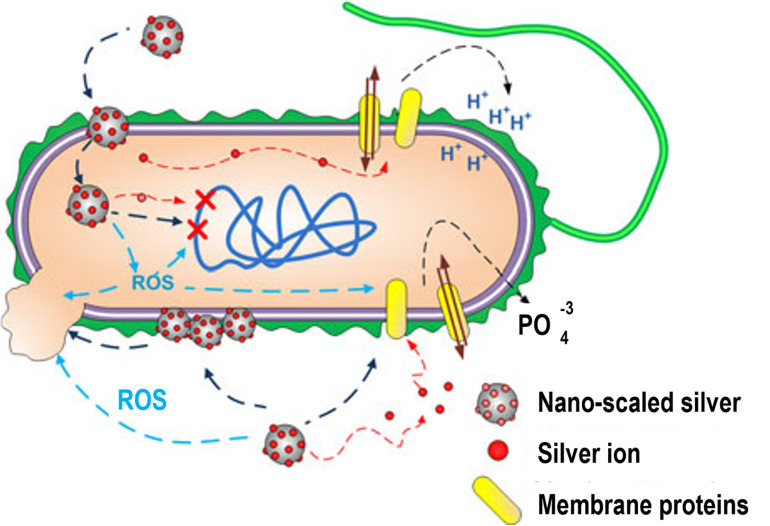
Ag NPs interaction with bacterial cells. Ag NPs can induce (1) Ag ions leakage and ROS generation, (2) membrane proteins dysfunction, (3) accumulation in cell membrane influencing the membrane permeability, (4) DNA damage. Adapted from Marambio-Jones and Hoek (2010) with permission.

**FIGURE 7:**
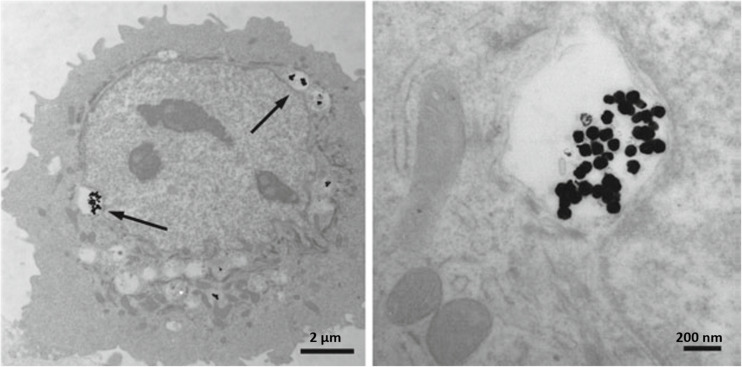
Transmission electron micrographs depicting Ag NPs uptake into HEK cells. **(A)** 80 nm Ag NPs within cytoplasmic vacuoles of a HEK; **(B)** higher magnification of the **(A)**. Arrows point to Ag NPs. Adapted from Deyhle et al. (2012).

The dose of Ag NPs utilization for inhibition of microbial growth must be kept under the range of human cell cytotoxicity. *In vitro* cell studies indicated the dependence of cytotoxicity on the size of Ag NPs and related ROS generation in different cell lines including fibrosarcoma, skin carcinoma, fibroblast, glioblastoma, hepatoma, alveolar, and keratinocyte (Wijnhoven et al., 2009; Samberg et al., 2010). In addition to size, the cytotoxicity and genotoxicity of Ag NPs are associated with its coating, concentration, exposure time, environmental factors, particle aggregation, surface oxidation to form silver oxides, etc. (Akter et al., 2018). Both Ag and its oxides have the potential to release Ag^+^ and Ag^0^ into media which consequently results in ionic Ag concentration in the environmental media and causes some degree of dysfunctions in mitochondria (Reidy et al., 2013). Subsequently, the interaction of Ag NPs with cell membrane proteins can lead to activation of signaling pathways for ROS generation and eventually cause proteins and nucleic acids destruction because of the potent affinity of silver for sulfur, at the end all of these events led to apoptosis and reductions in cell proliferation (Haase et al., 2012). Figure 7 shows the TEM images indicating AG NPs uptake in HEK cells.

There are some limited *in vivo* studies about the Ag NPs toxicity, it was shown that these NPs induced some harmful impacts on reproduction, malformations, and various morphological destructions in different animal models (Zhang X.F. et al., 2015). Drinking Ag NPs contained water to rats for 1–2 weeks duration, indicated Ag NPs distribution in musculus soleus, cerebellum, spleen, duodenum, and myocardial muscle (Pelkonen et al., 2003), also a dose-dependent Ag NPs accumulation in the liver of rats was reported (Kim et al., 2008). Prolonged intake of Ag NPs in the salt form with low concentrations led to fatty degeneration in the liver and kidneys together with variations in blood cells (Wijnhoven et al., 2009). Intravenous Ag NPs injection in rats showed 40 mg/kg dose (higher than 20 mg/kg dose values considered as toxic in rat models) can cause a considerable increase in liver enzymes whereas ROS increasing was detected in blood serum also TEM micrographs indicated the particle deposition in the liver and kidney of rats (Tiwari et al., 2011).

### Gold Nanoparticles and Their Toxicity

Gold (Au) NPs are also having a place between widespread NPs since they can be used in order to evaluate the cellular uptake and tissue distribution of particles, due to their easy to detect nature by electron microscopy and it has other applications as listed in Table 1. In addition, gold salts such as sodium gold thiomalate are utilized as decisive disease-modifying antirheumatic agents (Fadeel and Garcia-Bennett, 2010) but its long-term accumulation in the body can cause cytotoxic effects.

It was confirmed that the cellular response to Au NPs is size-dependent. For instance, 1.4 nm Au NPs is among the most toxic conditions of these NPs and results in rapid cell death by necrosis (Pan et al., 2007) while it seems to be non-toxic in 15 nm condition (Chen et al., 2009). *In vitro* studies about the Au NPs (35 nm) indicated its low toxicity for murine RAW macrophages with no considerable cell functionality blockage (Shukla et al., 2005). Coradeghini et al. (2013) investigated the effect of 5–20 nm Au NPs on human fetal lung fibroblast cells (MRC-5) and no considerable effect on the viability of MRC-5 cells was detected but cell proliferation was inhibited. Also, the oxidative DNA damage was confirmed due to NPs’ destructive effects on DNA. The smaller the Au NPs, the higher its tendency to induce toxicity since smaller NPs can easily bind on cellular surfaces. For example, Au NPs with 1.4 nm diameter are capable to bind with DNA and influence genes (mutation) compared to their larger counterparts (Yah, 2013). The dose of NPs has a crucial role in cytotoxicity, for example, Au NPs with a size range of 2–40 nm are biologically safe to MRC-5 cells but in exceeded dosage range (10 ppm dosage) apoptosis and up-regulated expression of pro-inflammatory genes and tumor necrosis was reported (Yen et al., 2009).

**FIGURE 8:**
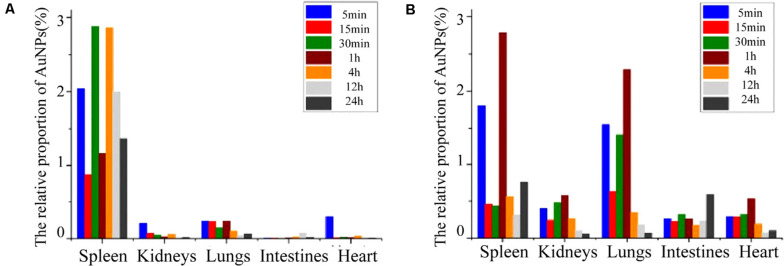
The relative distribution proportion of Au NPs in the spleen, kidneys, lungs, intestines, and heart of SD rats at 5, 15, 30 min, 1, 4, 12, and 24 h after IVI **(A)**, and after ISI in the tarsal tunnel **(B)**. Adapted from Shi et al. (2016) and Jia et al. (2017) with permission.

The Au NPs (5 nm) can preferentially bind to specified growth factors like vascular endothelial growth factor (VEGF) perhaps by cysteine residues of the heparin-binding domain and cause the inhibition of angiogenesis in a mouse model (Fadeel and Garcia-Bennett, 2010). Intravenous implementation of Au NPs (18 nm diameter) in rats showed that NPs were selectively accumulated within the liver and spleen, while NPs with 1 nm size were secreted in urine and feces. In addition, 3.7% of 1 nm sized particles remained in the blood at the first 24 h hence Au NPs interaction is size-dependent (Semmler-Behnke et al., 2008). Sonavane et al. (2008) studied the tissue distribution of Au NPs in rats, NPs intravenous exposure confirmed the highest accumulation in the liver and with lower amounts in the lung, kidney, and spleen. The smaller NPs with 15 and 50 nm size even can be found in the brain which indicates its ability to pass the blood-brain barrier hence they have the potential to get into the brain through neuronal transport. Au NPs with 20 nm size entry via inhalation can concentrate in the olfactory bulb of rats (Yu et al., 2007). Figure 8 shows the relative distribution proportion of the Au NPs in the various organs of rats including spleen, kidneys, lungs, intestines, and heart at different time durations.

**FIGURE 9:**
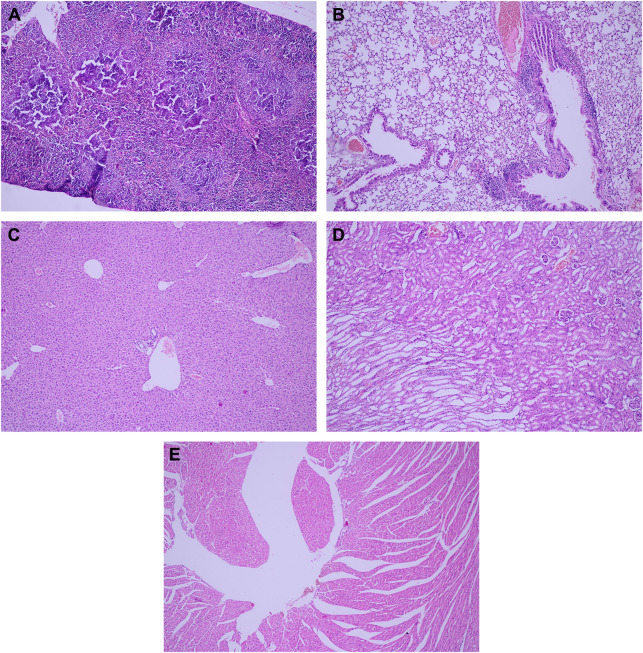
The pathology examination of the Au NPs treated rat organs, in 1,100 μg/kg dosage the pathology examination of the organs showed no degenerative, inflammatory, vascular, necrotic, or apoptotic lesions over the spleen **(A)**, lung **(B)**, liver **(C)**, kidney **(D)**, and heart **(E)**. Adapted from Berce et al. (2016) with permission from Dove Medical Press.

**FIGURE 10:**
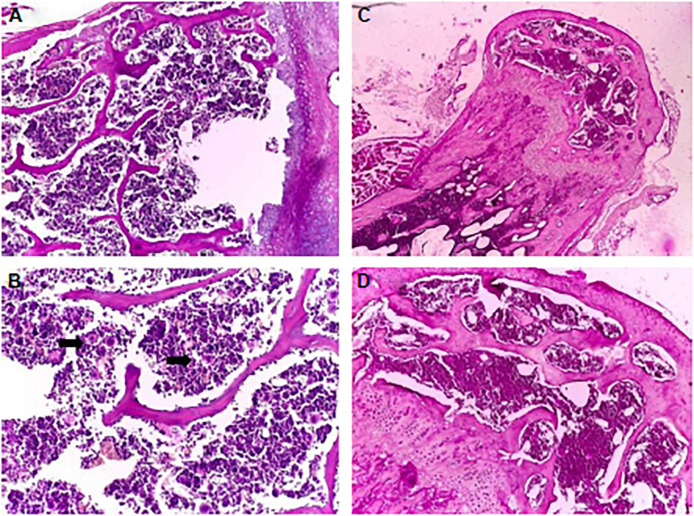
The pathology experiment of the bone marrow of the mice treated with Tween 20-GNPs **(A,B)** and of the control group **(C,D)**. In **(A,C)** the magnification was ×4, and in **(B,D)** ×20 magnification was used. The black arrows indicate the megakaryocytes. Adapted from Berce et al. (2016) with permission from Dove Medical Press.

Berce et al. (2016) investigated the bone marrow toxicity of Au NPs in rats, it was shown that Au NPs accumulated in the hematopoietic bone tissue and unfortunately resulting in severe side effects such as leucopoiesis and megakaryopoiesis and also increased levels of white blood cell and platelet were found in Au NPs treated rats compared to control ones, indicating its toxic effects. Figure 9 shows the pathology examination of the Au NPs treated rat organs as it was seen in the 1,100 μg/kg dosage no considerable sign of degenerative, inflammatory, vascular, necrotic, or apoptotic lesions was detected. While the pathology experiments through bone marrow and sternum in Figure 10 showed that the mice which got the daily iv Tween^®^ 20-GNPs had increased megakaryopoiesis as opposed to the control group. Figures 10A,B shows the increased megakaryopoiesis as opposed to the control group in Figures 10C,D, but no bone marrow fibrosis was detected.

### Zinc and Zinc Oxide Cytotoxicity

The zinc and zinc oxides were listed as safe substances in a US Food and Drug Administration (USFDA) (U.S. Food Drug Administration, 2019), while in the NPs condition it can induce toxicity into the surrounding environment. *In vitro* toxicity investigations of ZnO NPs in the size range of 40–48 nm in exposure to Chlorella Vulgaris indicated the reduction in viability, superoxide dismutase (SOD), and glutathione (GSH) and also increment of lactate dehydrogenase (LDH) (Suman et al., 2015). This finding indicates the considerable impact of ROS production in the cytotoxicity of ZnO NPs. Together with shape and concentration, the surface charge of ZnO NPs has a key role in its toxicity. It was believed that the positively charged NPs induce more toxicity and it can affect the cellular uptake and intracellular location (Asati et al., 2010; De Angelis et al., 2013). Kim et al. (2014) also proved the higher cytotoxicity of positively charged ZnO NPs in comparison to the negatively charged ones. In addition, genotoxicity and DNA damage was seen in ZnO treated MRC5 lung cells along with the high secretion of extracellular LDH and reduction in cell viability (Ng et al., 2017). Also, the Zn^2+^ release in ZnO NPs could lead to free radical emissions from the NPs surface and resulted in metabolic disbalance and fluctuation in ionic state of cells related to the deterrence of ion transport and defects in ionic homeostasis (Namvar et al., 2015; Suman et al., 2015).

ZnO NPs treatment of rats with 300 mg/kg dose showed the NPs concentration in the liver which led to cell trauma also a considerable DNA lesion in the liver was seen which resulting in oxidative stress caused DNA damage (Sharma et al., 2012), Figure 11 shows the pathological alterations in the liver and kidney of rats, treated with ZnO NPs for 14 consecutive days and also the control samples. The intraperitoneal injection with 50–200 mg of ZnO NPs/kg body dosage in Wistar rats indicated the dose-dependent toxicity behavior of ZnO NPs with considerable ROS generation also a major enhancement in liver enzymes at the concentration of 100 mg/kg animal body weight was reported (Abbasalipourkabir et al., 2015). ZnO NPs exposed liver tissue of animals indicated inflammation, increased congestion, chromatin condensation, and apoptosis, the tissue distribution analysis of ZnO NPs confirmed the increasing zinc dosages in the liver, large intestine, small intestine, and feces and some degree of hyperkeratosis and papillomatosis were detected in the skin (Ryu et al., 2014). Hence, ZnO NPs have toxic effects in both *in vitro* and *in vivo* studies including cytotoxicity, oxidative stress, and genotoxicity thus exposure to ZnO NPs should be considered and controlled precisely.

**FIGURE 11:**
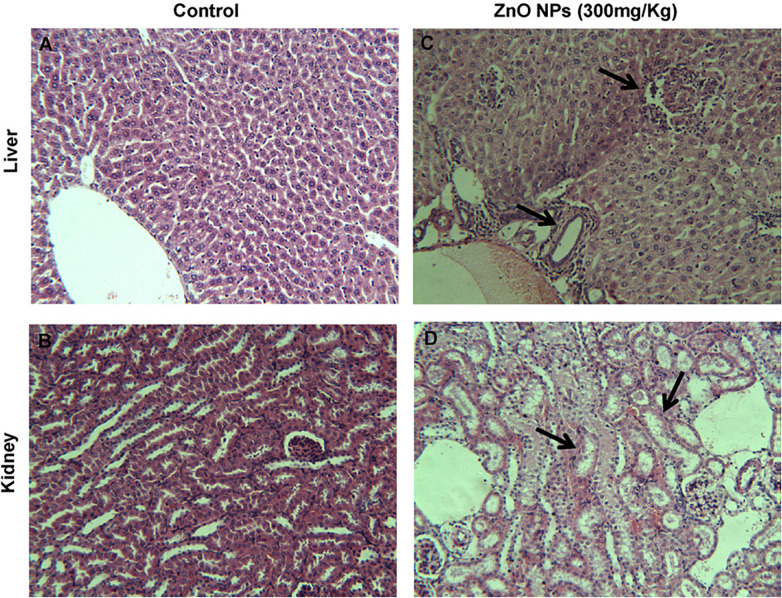
Histopathology of liver and kidney tissues in mice, ZnO NPs treated for 14 consecutive days. **(A,B)** Control group showing normal liver and kidney, **(C,D)** pathological alterations in the liver and kidney of ZnO NPs (300 mg/kg) treated group (indicated by arrow); magnification (200×). Adapted from Sharma et al. (2012) with permission.

### Toxicity of Copper and Copper Oxide NPs

Copper oxide (CuO) NPs have special characteristics like spin dynamics, high-temperature superconductivity, and electron correlation effects (El-Trass et al., 2012). Copper can be found in two ionic conditions Cu^1+^ and Cu^2+^ hence it can be interacting with biochemical reactions both as a reducing or oxidizing agent, nevertheless, it is not favorable from the toxicity aspect since copper ions are capable to induce oxidative stress (Valko et al., 2005), genotoxicity (Adeyemi et al., 2020), and free radical production (Fahmy et al., 2020).

**FIGURE 12:**
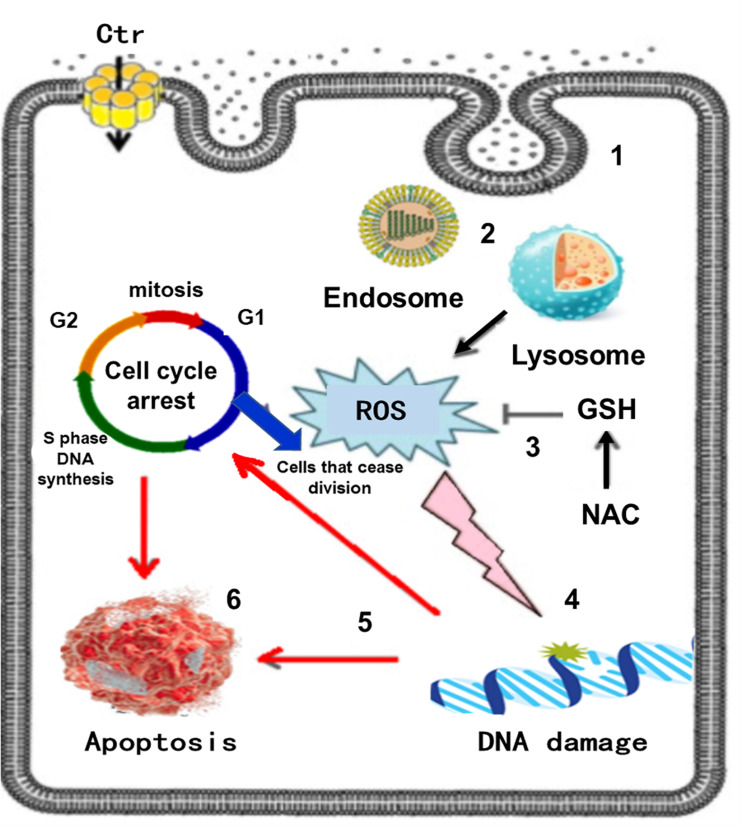
*In vitro* toxicity model of CuO NPs, the sequence of events in Poly toxicity. **(1)** CuO NPs are taken up via endocytosis, **(2)** Endocytotic vesicles are converted to lysosomes via autophagy, **(3)** ROS generation, other molecules can be oxidized mitigated by the antioxidant GSH and its precursor NAC, **(4)** ROS attacks DNA in the nucleus, **(5)** DNA damage activates signaling systems that induce cell cycle arrest, **(6)** Cell death by apoptosis. Reproduced from Thit et al. (2015) with permission.

*In vitro* examinations on the toxicity of CuO NPs on human breast cancer MCF-7 cells had shown some morphological changes in cells, also autophagic vacuoles were detected and the cell cycle arrest caused apoptosis (Laha et al., 2014). The study about lung epithelial cells treated with CuO NPs with 9.2 nm size with at various concentrations indicated that these NPs led to a reduction of cell cytotoxicity and increased level of dose-dependent oxidative stress (Jing et al., 2015). The effect of size and shapes on the toxicity of CuO NPs was investigated by Thit et al. (2015), two sizes of CuO NPs with 6 nm and 100 nm larger polydispersed CuO NPs, also microparticles and Cu ions were examined in epithelial kidney cells. The most toxic state belongs to the polydispersed CuO NPs and they induced a considerable increment in intracellular ROS generation, DNA damage, and cell death, Figure 12 schematically presents the *in vitro* toxicity model of CuO NPs.

*In vivo* studies in mouse models have shown that CuO NPs induced obviously epigenetic changes (Lu et al., 2016). Oral exposure of CuO NPs into rats showed the NP uptake in spleen, liver, kidney, brain, blood, lung, heart, urine, and feces (Lee et al., 2016). The CuO NPs exposed rats for up to 26 days showed some signs of increased Alanine Aminotransferase (ALT) levels as a liver damage index, also in 512 mg/kg dosage, no variations of histopathology were detected in liver, bone marrow, and stomach. The released Cu ions interfered with the immune system by lymphoid cell depletion in thymus and spleen organs, it should be said that the dissolution and biodistribution of NPs have a potential to act as a key factor in the toxic behavior of CuO treated samples (De Jong et al., 2019). Other studies in relation to the toxicity issues of metal and metal oxide NPs are listed in Table 3.

**TABLE 3:** *In vitro* and *in vivo* nanotoxicology studies of metal and metal oxide nanoparticles.

Material	Condition	Properties	Cell line or animal model	Conclusion	References
**Ag**	*In vitro*	20–30 nm	Caco-2, SW480	No significant ROS generation, increased inflammation, increased cell death and cell stress.	Abbott Chalew and Schwab, 2013
		Two nano-sized and two microsized	Human red blood cells	NPs were more hemolytic than micron-sized particles at equivalent mass concentrations > 220 μg/ml and at surface area concentrations > 10 cm (2)/ml, NPs released more Ag ions than microsized particles.	Choi et al., 2011
	*In vivo*	Silver-coated wound dressing Acticoat (1 week)	Human burns patient	Hepatotoxicity and argyria-like symptoms, Ag increase in plasma, urine and liver enzymes.	Trop et al., 2006
		30, 300 or 1000 mg/kg/day for 28 days (60 nm in size) per oral	Sprague- Dawley rats	Higher than 300 mg of NPs may result in slight liver damage, do not induce genetic toxicity, a gender-related difference in the accumulation of silver was noted in the kidneys, with a twofold increase in the female kidneys.	Kim et al., 2008
**Al**	*In vitro*	<500 nm	A549, THP-1	Low toxicity in MTT assay.	Lanone et al., 2009
**Ni**	*In vitro*	<500 nm	A549, THP-1	Low to moderate toxicity in MTT assay.	Lanone et al., 2009
**Co**	*In vitro*	<500 nm	A549, THP-1	Co NPs induced toxicity only when incorporated as a Nickel–Cobalt–Manganese mixed variant.	Lanone et al., 2009
**Au**	*In vitro*	Nanorods with 4:1 length-to-diameter ratio	HT29	Cytotoxicity caused by free CTAB, overcoating with polymer is useful.	Alkilany et al., 2009
	*In vivo*	8 mg/kg/week (3–100 nm in size) (4 weeks) intraperitoneal	BALB/C mice	NPs ranging from 8 to 37 nm induced severe sickness, fatigue, loss of appetite, change of fur color, and weight loss, from day 14 they exhibited a camel-like back and crooked spine. Pathological studies showed an increase of Kupffer cells in the liver, loss of structural integrity in the lungs, and diffusion of white pulp in the spleen.	Chen et al., 2009
		0.17, 0.85, and 4.26 mg/kg body weight (13 nm in size), (30 min after injection for 7 days) Intravenous, coated with PEG	BALB/C mice	Acute inflammation and apoptosis in the liver, NPs accumulate in the liver and spleen for up to 7 days with long blood circulation times, NPs presence in cytoplasmic vesicles and lysosomes of liver Kupffer cells and spleen macrophages.	Cho W.S. et al., 2009
		(12.5 nm in size) (40, 200, or 400 μg/kg/day for 8 days), intraperitoneal	C57/BL6 mice	NPs internalized inside the cell via a mechanism involving pinocytosis, also NPs internalization in lysosomal bodies arranged in perinuclear fashion, Au NPs were non-cytotoxic, non-immunogenic, and biocompatible properties.	Shukla et al., 2005
**Ti and TiO_2_**	*In vitro*	10-300 nm	Caco-2	DNA damage dependency on sample processing conditions, cytotoxic in LDH and WST-1 assay.	Gerloff et al., 2009
		21 nm	Caco-2, SW480	No significant ROS generation, increased inflammation, increased cell death and cell stress.	Abbott Chalew and Schwab, 2013
		21 nm	16HBE, A549	No considerable effect on 16-HBE or A549 cell viability, strong aggregation in culture media.	Guadagnini et al., 2015
		<500 nm	A549, THP-1	Moderate toxicity in MTT assay.	Lanone et al., 2009
	*In vivo*	NPs containing sunscreen, mean particle size of 20 nm	Human volunteers	NPs penetrate deeper into human skin from an oily dispersion than from an aqueous one.	Bennat and Müller-Goymann, 2000
**ZnO**	*In vitro*	20 nm	Caco-2, SW480	Toxic but no significant ROS generation, increased inflammation, increased cell death and cell stress.	Abbott Chalew and Schwab, 2013
		10–20 nm	Caco-2	DNA damage, cytotoxic in LDH and WST-1 assay.	Gerloff et al., 2009
		<500 nm	A549, THP-1	High toxicity in MTT assay.	Lanone et al., 2009
		288.2 and 265.7	Alveolar type II epithelial cells Ñ10	Oxidative stress generation induced by Zn ions, Decrease in cell viability after 6 and 24 h of incubation.	Xie et al., 2012
	*In vivo*	Coated and uncoated NPs with 74.0 nm and 65.0 nm size	Human volunteers	NPs did not enter or cause cellular toxicity in the viable epidermis, Zinc ion concentrations slightly increased, repeated application of ZnO NPs to the skin, as used in sunscreen products was determined as safe.	Mohammed et al., 2019
**MgO**	*In vitro*	8 nm	Caco-2	No cytotoxicity in LDH and WST-1 assay.	Gerloff et al., 2009
**SiO and SiO_2_**	*In vitro*	14 nm	Caco-2	Glutathione depletion, DNA damage, cytotoxic in LDH and WST-1 assay.	Gerloff et al., 2009
		25 and 50 nm, modified and not modified with sodium oleate	16HBE, A549	Dose, time and size dependent effects, 25 nm NPs are more toxic than 50 nm ones at lower concentrations, ROS generation ROS at toxic concentrations.	Guadagnini et al., 2015
		100 nm	HeLa, 3T3	Cell viability and survival decreased only about 20% at high concentration of 100 μg/mL, no significant toxic effects.	Xia et al., 2013
	*In vivo*	20 mg/animal (1 or 2 months), intratracheal instillation	Wistar rats	Changes in pathology and fibrotic grade, the lung/body coefficient and hydroxyproline content of SiO_2_ NPs were lower than microsized SiO_2_.	Chen et al., 2004
		50 mg/kg (50, 100 or 200 nm in size), (12, 24, 48 and 72 h, 7 days) intravenous	BALB/C mice	NPs trapped by macrophages in the spleen and liver and remained there until 4 weeks after the single injection, Macrophage mediated frustrated phagocytosis of larger NPs resulted in release of pro-inflammatory cytokines and cell infiltrates within hepatic parenchyma.	Cho M. et al., 2009
		2 mg/kg (20-25 nm in size) (24 h), intravenous	Nude mice	Higher accumulation of NPs in liver, spleen, and stomach than in kidney, heart, and lungs, hepatobiliary excretion of NPs after 15 days.	Kumar et al., 2010
**Cu, CuO and CuS**	*In vitro*	50 and 100 nm, surface charge	Caco-2	Positively charged NPs have higher toxicity and cell uptake, NPs transfer is a dynamin-dependent process.	Bannunah et al., 2014
		50 nm	A549, SAEC	Cell cycle arrest by Cu ions, highly toxic, inhibition of cell proliferation genes, apoptosis.	Hanagata et al., 2011
		<500 nm	A549, THP-1	High toxicity in MTT assay.	Lanone et al., 2009
		Length of 59.4 nm and thickness of 23.8 nm	HUVECs, RAW 264.7, KB, HeLa	Cell viability reduction in HUVECs at higher than 100 μg/mL dosages, toxicity to HUVEC and RAW 264.7 cells, NPs uptake in RAW 264.7 cells, no considerable change in cytoskeleton components.	Feng et al., 2015
	*In vivo*	Micro-Cu (1 μm), and nano-Cu (80–100 nm),	Sprague-Dawley rats	Cu NPs changed the immune function of the spleen, Alteration in the number of blood cells in rats and lymphocyte subpopulation in the spleen, antibody production and obvious histopathology changes.	Zhou et al., 2019
**FeO and Fe_3_O_4_**	*In vitro*	10 nm, without and with polyethylenoxide (PEO) coating	PC3, C4-2, HUVECs	Viability reduction, coated NPs uptake by cells, the surface-modified NPs are more toxic than NPs without shells.	Häfeli et al., 2009
		8 nm, modified and not modified with sodium oleate	16HBE, A549	Sodium oleate coating led to an increase in cytotoxicity, strong aggregation in culture media, toxic and inducing cytotoxicity in a dose, time and coating dependent manner.	Guadagnini et al., 2015
	*In vivo*	Ferrite and manganese ferrite oxide with sizes between 3 and 20 nm	Zebrafish embryos and mice	In manganese-based NPs concentrations above 100 μg/mL showed a low survival rate (<50%), absence of toxicity in mice	Caro et al., 2019
**CeO_2_**	*In vitro*	15, 25, 30, and 45 nm	BEAS- 2B	ROS generation led to cell death, NPs absorption by cells and localized in the perinuclear space	Park et al., 2008

### Toxicity Prevention in Metallic and Metal Oxide NPs

The size, morphology, concentration, aggregation mode, charge, surface properties all have an impact on toxicity and must be considered in order to prevent the harmful effects of NPs. It was known that toxicity of metallic and metal oxide NPs is directly related to its surface properties hence alterations in the surface of these NPs can be a good idea for mitigating their possible harmful effects. In this regard, various safe surface designs are the spotlights and can be listed as utilization of surface coatings (Osmond-McLeod et al., 2013), core-shell structures (Davidson et al., 2015), doping-based methods (Wang et al., 2012), geometric control (Ji et al., 2012), and surface passivation methods (Cai et al., 2017). Among these methods, the coating approaches seem to be simpler and more controllable and it is almost applicable to every metallic NPs. These methods can affect the surface reactivity and ion outlet in order to avoid any cytotoxic occurrence. One of the coating and surface passivation methods is sulfidation, for example, the existence of sulfide can avoid AgNO_3_ toxicity (Bowles et al., 2002). Also, Levard et al. (2013) reported that sulfidation of silver NPs can hinder Ag ion mobility and reduce its dissolution rate. Considering gastrointestinal digestion impact, Martirosyan et al. (2014) used food matrix component phenolic compounds (PCs) to prevent from the toxicology of Ag NPs. In this regard, two major factors involving in the toxicity of Ag NPs (the release of Ag^+^ and ROS) were studied. Results showed that two PCs, quercetin and kaempferol, relatively defended the Caco-2 cells from Ag NPs induced toxicity and these PCs protected the epithelial barrier integrity which disrupted by NPs. Future investigations seem to be necessary to find more sophisticated methods in order to precise and complete toxicity prevention.

## Conclusion

Nanotechnology as one of the exciting and modern branches of science found a lot of applications in various technologies from the food and cosmetic industry to medicine and agriculture, hence humanity is in direct contact with these nanoparticles (NPs). Although, the nanosized materials have many benefits compared to coarse sizes they can also have unfavorable effects since they have the potential to pass the natural barriers of live cells and tissues and cause toxic and inflammatory issues. Because of these problems, a new branch of science entitled nanotoxicology has emerged with the aim to elucidate the possible effects of NPs and the related parameters affecting the cytotoxicity of nanomaterials. Metal and metal oxide materials are among the most used NPs so this review paper dedicated to analyze the key factors influencing the toxicity of these NPs and review the *in vitro* and *in vivo* studies to find out the possible hazards of NPs and found a detailed guideline to control and decrease the adverse effects of metal and metal oxide NPs. In order to attain this goal, firstly the toxicity mechanisms including reactive oxygen species (ROS) generation, the effect of NPs on cell membranes, cytoskeleton components, DNA, mitochondria, and lysosomes was discussed. Secondly, because of the obvious effects of physical and chemical characteristics on toxicity, they were carefully reviewed. It was found that the size of NPs has a significant effect since its nano-sized range by increasing the specific surface area led to more cellular interactions and toxicity. The other key factors affecting the cytotoxicity of NPs are shape and dimensionality, chemical composition and NPs concentration or dosage, crystalline structures, solubility, hydrophilicity, surface charge, and agglomeration condition. In this regard, it was believed that it is critical to control the physicochemical properties of NPs in order to achieve more safe and reliable NPs since even natural non-toxic and even antibacterial materials such as Ag, Cu, Ti, and Zn can induce toxicity in some ranges of size, dosage and surface charges. Overall, it seems that violating the cell passage system for example by decreasing the size of NPs to smaller than cellular subunits, organelles, and cells and letting them permeate and enter into the biological structures should be strictly prevented. Thirdly, some highlight findings of *in vitro* and *in vivo* studies about the toxicity of metal and metal oxide NPs were discussed to determine key factors. In the end, it is hoped that increasing the awareness and information about this subject opens a new horizon to understand more about the nanotoxicology and design the modern materials and procedures with the safe thresholds. These modern NPs should be designed meticulously by taking into account all the intriguing and complex aspects that arise from nanometric size ranges and also the other affecting factors.

The emerging trends and prospects in metallic NPs’ toxicity studies are quite broad including modern NPs designs with optimal properties, enhancing their favorable effects and minimizing the potential toxicity, detection of toxicity transmitting species and targets by considering their life cycle, incorporation of various coating and surface treatments to decrease the harmful results while maintaining the favorable properties. Many aspects of these issues are still unsolved and need further studies in the future to overcome the toxicity limitations of metallic NPs and other present to-date barriers. In this regard, methods based on the simultaneous use of NPs with antitoxic strategies seem to be more promising. Also, in future studies, more attention should be paid to possible effects of biological fluids and surrounding tissues, biokinetics, involved mechanisms, and other chemical and biological factors. Moreover, there is a demand for more sophisticated and validated *in vitro* models that are prognostic of *in vivo* experiments outcomes. Finally, the resultant guidelines should have a potency to underlie the exact NPs’ interactions with biological systems in order to support a complete correct risk assessment. Hence, various scientific disciplines including chemistry, physics, medicine, and biology should be involved and interact together to shade light on all the complex cellular-molecular events.

## Author Contributions

SA wrote the main part of the manuscript. QW and JuY greatly contributed to the titanium and copper parts. ME made major contributions particularly in choosing the figures. SA, JiY, ME, and JL made significant contribution to the revision stage. YT and SA prepared and formulated the references. All the authors contributed to the article and approved the submitted version.

## Conflict of Interest

The authors declare that the research was conducted in the absence of any commercial or financial relationships that could be construed as a potential conflict of interest.

## References

[B1] Abbasalipourkabir R. Moradi H. Zarei S. Asadi S. Salehzadeh A. Ghafourikhosroshahi A. (2015). Toxicity of zinc oxide nanoparticles on adult male Wistar rats. *Food Chem. Toxicol.* 84 154–160. 10.1016/j.fct.2015.08.019 26316185

[B2] Abbott Chalew T. E. Schwab K. J. (2013). Toxicity of commercially available engineered nanoparticles to Caco-2 and SW480 human intestinal epithelial cells. *Cell Biol. Toxicol.* 29 101–116. 10.1007/s10565-013-9241-6 23468361

[B3] Abdelgied M. El-Gazzar A. M. Alexander D. B. Alexander W. T. Numano T. Iigou M. (2019). Pulmonary and pleural toxicity of potassium octatitanate fibers, rutile titanium dioxide nanoparticles, and MWCNT-7 in male Fischer 344 rats. *Arch. Toxicol.* 93 909–920. 10.1007/s00204-019-02410-z 30759267

[B4] Adeyemi J. A. Machado A. R. T. Ogunjimi A. T. Alberici L. C. Antunes L. M. G. Barbosa F. (2020). Cytotoxicity, mutagenicity, oxidative stress and mitochondrial impairment in human hepatoma (HepG2) cells exposed to copper oxide, copper-iron oxide and carbon nanoparticles. *Ecotoxicol. Environ. Saf.* 189:109982. 10.1016/j.ecoenv.2019.109982 31830603

[B5] Akter M. Sikder M. T. Rahman M. M. Ullah A. K. M. A. Hossain K. F. B. Banik S. (2018). A systematic review on silver nanoparticles-induced cytotoxicity: physicochemical properties and perspectives. *J. Adv. Res.* 9 1–16. 10.1016/j.jare.2017.10.008 30046482 PMC6057238

[B6] Alkilany A. M. Nagaria P. K. Hexel C. R. Shaw T. J. Murphy C. J. Wyatt M. D. (2009). Cellular uptake and cytotoxicity of gold nanorods: molecular origin of cytotoxicity and surface effects. *Small* 5 701–708. 10.1002/smll.200801546 19226599

[B7] Ansarian I. Shaeri M. H. Ebrahimi M. Minárik P. Bartha K. (2019). Microstructure evolution and mechanical behaviour of severely deformed pure titanium through multi directional forging. *J. Alloys Compd.* 776 83–95. 10.1016/j.jallcom.2018.10.196

[B8] Asati A. Santra S. Kaittanis C. Perez J. M. (2010). Surface-charge-dependent cell localization and cytotoxicity of cerium oxide nanoparticles. *ACS Nano* 4 5321–5331. 10.1021/nn100816s 20690607 PMC2947560

[B9] ASTM (2012). *ASTM E2456-06, Standard Terminology Relating to Nanotechnology.* West Conshohocken, PA: ASTM International.

[B10] Attarilar S. Djavanroodi F. Irfan O. M. Al-Mufadi F. A. Ebrahimi M. Wang Q. D. (2020). Strain uniformity footprint on mechanical performance and erosion-corrosion behavior of equal channel angular pressed pure titanium. *Results Phys.* 17:103141 10.1016/j.rinp.2020.103141

[B11] Attarilar S. Salehi M. T. Al-Fadhalah K. J. Djavanroodi F. Mozafari M. (2019). Functionally graded titanium implants: characteristic enhancement induced by combined severe plastic deformation. *PLoS One* 14:e0221491. 10.1371/journal.pone.0221491 31442256 PMC6707610

[B12] Bannunah A. M. Vllasaliu D. Lord J. Stolnik S. (2014). Mechanisms of nanoparticle internalization and transport across an intestinal epithelial cell model: effect of size and surface charge. *Mol. Pharm.* 11 4363–4373. 10.1021/mp500439c 25327847

[B13] Ben Younes N. R. Amara S. Mrad I. Ben-Slama I. Jeljeli M. Omri K. (2015). Subacute toxicity of titanium dioxide (TiO2) nanoparticles in male rats: emotional behavior and pathophysiological examination. *Environ. Sci. Pollut. Res.* 22 8728–8737. 10.1007/s11356-014-4002-5 25572266

[B14] Bennat C. Müller-Goymann C. C. (2000). Skin penetration and stabilization of formulations containing microfine titanium dioxide as physical UV filter. *Int. J. Cosmet. Sci.* 22 271–283. 10.1046/j.1467-2494.2000.00009.x 18503414

[B15] Berce C. Lucan C. Petrushev B. Boca S. Miclean M. Sarpataki O. (2016). In vivo assessment of bone marrow toxicity by gold nanoparticle-based bioconjugates in CrL:CD1(ICR) mice. *Int. J. Nanomedicine* 11 4261–4273. 10.2147/IJN.S108711 27621620 PMC5012637

[B16] Bowles K. C. Bianchini A. Brauner C. J. Kramer J. R. Wood C. M. (2002). Evaluation of the effect of reactive sulfide on the acute toxicity of silver (I) to Daphnia magna. Part 1: description of the chemical system. *Environ. Toxicol. Chem.* 21 1286–1293. 12069316

[B17] Bressan E. Ferroni L. Gardin C. Rigo C. Stocchero M. Vindigni V. (2013). Silver nanoparticles and mitochondrial interaction. *Int. J. Dentistry* 2013 1–8. 10.1155/2013/312747 PMC3786470 24101927

[B18] Brooker R. J. (2018). *Genetics: Analysis & Principles*, 6th Edn New York, NY: McGraw-Hill Science.

[B19] Buzea C. Pacheco I. I. Robbie K. (2007). Nanomaterials and nanoparticles: sources and toxicity. *Biointerphases* 2 MR17–MR71. 10.1116/1.2815690 20419892

[B20] Cai X. Lee A. Ji Z. Huang C. Chang C. H. Wang X. (2017). Reduction of pulmonary toxicity of metal oxide nanoparticles by phosphonate-based surface passivation. *Part Fibre Toxicol.* 14 1–11. 10.1186/s12989-017-0193-5 28431555 PMC5399805

[B21] Caro C. Egea-Benavente D. Polvillo R. Royo J. L. Pernia Leal M. García-Martín M. L. (2019). Comprehensive toxicity assessment of PEGylated magnetic nanoparticles for in vivo applications. *Colloids Surfaces B Biointerfaces* 177 253–259. 10.1016/j.colsurfb.2019.01.051 30763790

[B22] Champion J. A. Mitragotri S. (2006). Role of target geometry in phagocytosis. *Proc. Natl. Acad. Sci. U.S.A.* 103 4930–4934. 10.1073/pnas.0600997103 16549762 PMC1458772

[B23] Chen H. (2018). Metal based nanoparticles in agricultural system: behavior, transport, and interaction with plants. *Chem. Speciat. Bioavailab.* 30 123–134. 10.1080/09542299.2018.1520050

[B24] Chen Q. Wang N. Zhu M. Lu J. Zhong H. (2018). Redox Biology TiO 2 nanoparticles cause mitochondrial dysfunction, activate in fl ammatory responses, and attenuate phagocytosis in macrophages: a proteomic and metabolomic insight. *Redox Biol.* 15 266–276. 10.1016/j.redox.2017.12.011 29294438 PMC5752088

[B25] Chen Y. Chen J. Dong J. Jin Y. (2004). Comparing study of the effect of nanosized silicon dioxide and microsized silicon dioxide on fibrogenesis in rats. *Toxicol. Ind. Health* 20 21–27. 10.1191/0748233704th190oa 15807405

[B26] Chen Y.-S. Hung Y.-C. Liau I. Huang G. S. (2009). Assessment of the in vivo toxicity of gold nanoparticles. *Nanoscale Res. Lett.* 4 858–864. 10.1007/s11671-009-9334-6 20596373 PMC2894102

[B27] Cho M. Cho W.-S. Choi M. Kim S. J. Han B. S. Kim S. H. (2009). The impact of size on tissue distribution and elimination by single intravenous injection of silica nanoparticles. *Toxicol. Lett.* 189 177–183. 10.1016/j.toxlet.2009.04.017 19397964

[B28] Cho W.-S. Cho M. Jeong J. Choi M. Cho H.-Y. Han B. S. (2009). Acute toxicity and pharmacokinetics of 13 nm-sized PEG-coated gold nanoparticles. *Toxicol. Appl. Pharmacol.* 236 16–24. 10.1016/j.taap.2008.12.023 19162059

[B29] Choi J. Reipa V. Hitchins V. M. Goering P. L. Malinauskas R. A. (2011). Physicochemical characterization and in vitro hemolysis evaluation of silver nanoparticles. *Toxicol. Sci.* 123 133–143. 10.1093/toxsci/kfr149 21652737

[B30] Coradeghini R. Gioria S. García C. P. Nativo P. Franchini F. Gilliland D. (2013). Size-dependent toxicity and cell interaction mechanisms of gold nanoparticles on mouse fibroblasts. *Toxicol. Lett.* 217 205–216. 10.1016/j.toxlet.2012.11.022 23246733

[B31] Davidson D. C. Derk R. He X. Stueckle T. A. Cohen J. Pirela S. V. (2015). Direct stimulation of human fibroblasts by nCeO2 in vitro is attenuated with an amorphous silica coating. *Part. Fibre Toxicol.* 13:23. 10.1186/s12989-016-0134-8 27142434 PMC4855843

[B32] Dayem A. A. Hossain M. K. Bin Lee S. Kim K. Saha S. K. Yang G. (2017). The role of reactive oxygen species (ROS) in the biological activities of metallic nanoparticles. *Int. J. Mol. Sci.* 18 1–21. 10.3390/ijms18010120 28075405 PMC5297754

[B33] De Angelis I. Barone F. Zijno A. Bizzarri L. Russo M. T. Pozzi R. (2013). Comparative study of ZnO and TiO2 nanoparticles: physicochemical characterisation and toxicological effects on human colon carcinoma cells. *Nanotoxicology* 7 1361–1372. 10.3109/17435390.2012.741724 23078188

[B34] De Jong W. H. De Rijk E. Bonetto A. Wohlleben W. Stone V. Brunelli A. (2019). Toxicity of copper oxide and basic copper carbonate nanoparticles after short-term oral exposure in rats. *Nanotoxicology* 13 50–72. 10.1080/17435390.2018.1530390 30451559

[B35] De Jong W. H. Hagens W. I. Krystek P. Burger M. C. Sips A. J. A. M. Geertsma R. E. (2008). Particle size-dependent organ distribution of gold nanoparticles after intravenous administration. *Biomaterials* 29 1912–1919. 10.1016/j.biomaterials.2007.12.037 18242692

[B36] De Matteis V. Cascione M. Brunetti V. Toma C. C. Rinaldi R. (2016). Toxicity assessment of anatase and rutile titanium dioxide nanoparticles: the role of degradation in different pH conditions and light exposure. *Toxicol. Vitr.* 37 201–210. 10.1016/j.tiv.2016.09.010 27622577

[B37] Devasagayam T. P. A. Tilak J. C. Boloor K. K. Sane K. Ghaskadbi S. Lele R. (2004). Free radicals and antioxidants in human health: current status and future prospects. *J. Assoc. Physicians India* 52 794–804. 15909857

[B38] Deyhle H. Schulz G. Bert M. (2012). *Encyclopedia of Nanotechnology.* Berlin: Springer Netherlands. Dordrecht.

[B39] Dhall A. Self W. (2018). Cerium oxide nanoparticles: a brief review of their synthesis methods and biomedical applications. *Antioxidants* 7 1–13. 10.3390/antiox7080097 30042320 PMC6116044

[B40] Ding Z. Zhang C. Xie L. Zhang L.-C. Wang L. Lu W. (2016). Effects of friction stir processing on the phase transformation and microstructure of TiO2-compounded Ti-6Al-4V alloy. *Metall. Mater. Trans. A* 47 5675–5679. 10.1007/s11661-016-3809-8

[B41] Dröge W. (2002). Free radicals in the physiological control of cell function. *Physiol. Rev.* 82 47–95. 10.1152/physrev.00018.2001 11773609

[B42] El Badawy A. M. Silva R. G. Morris B. Scheckel K. G. Suidan M. T. Tolaymat T. M. (2011). Surface charge-dependent toxicity of silver nanoparticles. *Environ. Sci. Technol.* 45 283–287. 10.1021/es1034188 21133412

[B43] El-Trass A. ElShamy H. El-Mehasseb I. El-Kemary M. (2012). CuO nanoparticles: synthesis, characterization, optical properties and interaction with amino acids. *Appl. Surf. Sci.* 258 2997–3001. 10.1016/j.apsusc.2011.11.025

[B44] Fabian E. Landsiedel R. Ma-Hock L. Wiench K. Wohlleben W. van Ravenzwaay B. (2008). Tissue distribution and toxicity of intravenously administered titanium dioxide nanoparticles in rats. *Arch. Toxicol.* 82 151–157. 10.1007/s00204-007-0253-y 18000654

[B45] Fadeel B. Garcia-Bennett A. E. (2010). Better safe than sorry: understanding the toxicological properties of inorganic nanoparticles manufactured for biomedical applications. *Adv. Drug Deliv. Rev.* 62 362–374. 10.1016/j.addr.2009.11.008 19900497

[B46] Fahmy H. M. Ebrahim N. M. Gaber M. H. (2020). In-vitro evaluation of copper/copper oxide nanoparticles cytotoxicity and genotoxicity in normal and cancer lung cell lines. *J. Trace Elem. Med. Biol.* 60:126481. 10.1016/j.jtemb.2020.126481 32135445

[B47] Feng W. Nie W. Cheng Y. Zhou X. Chen L. Qiu K. (2015). In vitro and in vivo toxicity studies of copper sulfide nanoplates for potential photothermal applications, Nanomedicine Nanotechnology. *Biol. Med.* 11 901–912. 10.1016/j.nano.2014.12.015 25652899

[B48] Flores-López L. Z. Espinoza-Gómez H. Somanathan R. (2019). Silver nanoparticles: electron transfer, reactive oxygen species, oxidative stress, beneficial and toxicological effects. Mini review, *J. Appl. Toxicol.* 39 16–26. 10.1002/jat.3654 29943411

[B49] Fröhlich E. (2013). Cellular targets and mechanisms in the cytotoxic action of non-biodegradable engineered nanoparticles. *Curr. Drug Metab.* 14 976–988. 10.2174/1389200211314090004 24160294 PMC3822521

[B50] Gallud A. Klöditz K. Ytterberg J. Östberg N. Katayama S. Skoog T. (2019). Cationic gold nanoparticles elicit mitochondrial dysfunction: a multi-omics study. *Sci. Reportsreports* 9 1–19. 10.1038/s41598-019-40579-6 30867451 PMC6416392

[B51] García-Hevia L. Valiente R. Martín-Rodríguez R. Renero-Lecuna C. González J. Rodríguez-Fernández L. (2016). Nano-ZnO leads to tubulin macrotube assembly and actin bundling, triggering cytoskeletal catastrophe and cell necrosis. *Nanoscale* 8 10963–10973. 10.1039/C6NR00391E 27228212

[B52] Gea M. Bonetta S. Iannarelli L. Giovannozzi A. M. Maurino V. Bonetta S. (2019). Shape-engineered titanium dioxide nanoparticles (TiO2-NPs): cytotoxicity and genotoxicity in bronchial epithelial cells. *Food Chem. Toxicol.* 127 89–100. 10.1016/j.fct.2019.02.043 30849403

[B53] Georgantzopoulou A. Serchi T. Cambier S. Leclercq C. C. Renaut J. Shao J. (2016). Effects of silver nanoparticles and ions on a co-culture model for the gastrointestinal epithelium. *Part. Fibre Toxicol.* 13:9. 10.1186/s12989-016-0117-9 26888332 PMC4756536

[B54] Gerloff K. Albrecht C. Boots A. W. Frster I. Schins R. P. F. (2009). Cytotoxicity and oxidative DNA damage by nanoparticles in human intestinal Caco-2 cells. *Nanotoxicology* 3 355–364. 10.3109/17435390903276933 21034372

[B55] Gode C. Attarilar S. Eghbali B. Ebrahimi M. (2015). “Electrochemical behavior of equal channel angular pressed titanium for biomedical application,” in *AIP Conference Proceedings*, College Park, ML: AIP.

[B56] Gogoi S. K. Gopinath P. Paul A. Ramesh A. Ghosh S. S. Chattopadhyay A. (2006). Green fluorescent protein-expressing *Escherichia coli* as a model system for investigating the antimicrobial activities of silver nanoparticles. *Langmuir* 22 9322–9328. 10.1021/la060661v 17042548

[B57] Gopinath P. Gogoi S. K. Chattopadhyay A. Ghosh S. S. (2008). Implications of silver nanoparticle induced cell apoptosis for in vitro gene therapy. *Nanotechnology* 19:075104 10.1088/0957-4484/19/7/075104 21817629

[B58] Gu H. Ding Z. Yang Z. Yu W. Zhang W. Lu W. (2019). Microstructure evolution and electrochemical properties of TiO2/Ti-35Nb-2Ta-3Zr micro/nano-composites fabricated by friction stir processing. *Mater. Des.* 169:107680 10.1016/j.matdes.2019.107680

[B59] Guadagnini R. Moreau K. Hussain S. Marano F. Boland S. (2015). Toxicity evaluation of engineered nanoparticles for medical applications using pulmonary epithelial cells. *Nanotoxicology* 9 25–32. 10.3109/17435390.2013.855830 24286383

[B60] Guo D. Zhang J. Huang Z. Jiang S. Gu N. (2015). Colloidal silver nanoparticles improve anti-leukemic drug efficacy via amplification of oxidative stress. *Colloids Surf. B Biointerfaces* 126 198–203. 10.1016/j.colsurfb.2014.12.023 25576804

[B61] Gurr J. R. Wang A. S. S. Chen C. H. Jan K. Y. (2005). Ultrafine titanium dioxide particles in the absence of photoactivation can induce oxidative damage to human bronchial epithelial cells. *Toxicology* 213 66–73. 10.1016/j.tox.2005.05.007 15970370

[B62] Haase A. Rott S. Mantion A. Graf P. Plendl J. Thünemann A. F. (2012). Effects of silver nanoparticles on primary mixed neural cell cultures: uptake. Oxidative stress and acute calcium responses. *Toxicol. Sci.* 126 457–468. 10.1093/toxsci/kfs003 22240980 PMC3307608

[B63] Häfeli U. O. Riffle J. S. Harris-Shekhawat L. Carmichael-Baranauskas A. Mark F. Dailey J. P. (2009). Cell uptake and in vitro toxicity of magnetic nanoparticles suitable for drug delivery. *Mol. Pharm.* 6 1417–1428. 10.1021/mp900083m 19445482

[B64] Halamoda Kenzaoui B. Chapuis Bernasconi C. Guney-Ayra S. Juillerat-Jeanneret L. (2012). Induction of oxidative stress, lysosome activation and autophagy by nanoparticles in human brain-derived endothelial cells. *Biochem. J.* 441 813–821. 10.1042/BJ20111252 22026563

[B65] Hanagata N. Zhuang F. Connolly S. Li J. Ogawa N. Xu M. (2011). Molecular responses of human lung epithelial cells to the toxicity of copper oxide nanoparticles inferred from whole genome expression analysis. *ACS Nano* 5 9326–9338. 10.1021/nn202966t 22077320

[B66] He X. (2009). “Chapter 18 - integration of physical, chemical, mechanical, and biopharmaceutical properties in solid oral dosage form development,” in *Developing Solid Oral Dosage Forms*, eds Qiu Y. Chen Y. Zhang G. Liu G. Z. (San Diego: Academic Press), 407–441.

[B67] Heinemann D. Schomaker M. Kalies S. Schieck M. Carlson R. Escobar H. M. (2013). Gold nanoparticle mediated laser transfection for efficient sirna mediated gene knock down. *PLoS One* 8:e58604. 10.1371/journal.pone.0058604 23536802 PMC3594183

[B68] Horie M. Nishio K. Fujita K. Kato H. Nakamura A. Kinugasa S. (2009). Ultrafine NiO particles induce cytotoxicity in vitro by cellular uptake and subsequent Ni(II) release. *Chem. Res. Toxicol.* 22 1415–1426. 10.1021/tx900171n 19630433

[B69] Hoseinpour V. Ghaemi N. (2018). Green synthesis of manganese nanoparticles: applications and future perspective–A review. *J. Photochem. Photobiol. B Biol.* 189 234–243. 10.1016/j.jphotobiol.2018.10.022 30412855

[B70] Huang Y. W. Cambre M. Lee H. J. (2017). The toxicity of nanoparticles depends on multiple molecular and physicochemical mechanisms. *Int. J. Mol. Sci.* 18:2702. 10.3390/ijms18122702 29236059 PMC5751303

[B71] Huo S. Jin S. Ma X. Xue X. Yang K. Kumar A. (2014). Ultrasmall gold nanoparticles as carriers for nucleus-based gene therapy due to size-dependent nuclear entry. *ACS Nano* 8 5852–5862. 10.1021/nn5008572 24824865 PMC4076024

[B72] Ispanixtlahuatl-Meráz O. Schins R. P. F. Chirino Y. I. (2018). Cell type specific cytoskeleton disruption induced by engineered nanoparticles. *Environ. Sci. Nano* 5 228–245. 10.1039/c7en00704c

[B73] Ji Z. Wang X. Zhang H. Lin S. Meng H. Sun B. (2012). Designed synthesis of CeO 2 nanorods and nanowires for studying toxicological effects of high aspect ratio nanomaterials. *ACS Nano* 6 5366–5380. 10.1021/nn3012114 22564147 PMC3651271

[B74] Jia Y. P. Ma B. Y. Wei X. W. Qian Z. Y. (2017). The in vitro and in vivo toxicity of gold nanoparticles. *Chinese Chem. Lett.* 28 691–702. 10.1016/j.cclet.2017.01.021

[B75] Jimeno-Romero A. Oron M. Cajaraville M. P. Soto M. Marigómez I. (2016). Nanoparticle size and combined toxicity of TiO2 and DSLS (surfactant) contribute to lysosomal responses in digestive cells of mussels exposed to TiO2 nanoparticles. *Nanotoxicology* 10 1168–1176. 10.1080/17435390.2016.1196250 27241615

[B76] Jing X. Park J. H. Peters T. M. Thorne P. S. (2015). Toxicity of copper oxide nanoparticles in lung epithelial cells exposed at the air–liquid interface compared with in vivo assessment. *Toxicol. Vitr.* 29 502–511. 10.1016/j.tiv.2014.12.023 25575782 PMC4373347

[B77] Kanchi S. Ahmed S. (eds) (2018). *Green Metal Nanoparticles.* Hoboken, NJ: John Wiley & Sons, Inc.

[B78] Karlsson H. L. Cronholm P. Gustafsson J. Möller L. (2008). Copper oxide nanoparticles are highly toxic: a comparison between metal oxide nanoparticles and carbon nanotubes. *Chem. Res. Toxicol.* 21 1726–1732. 10.1021/tx800064j 18710264

[B79] Katerji M. Filippova M. Duerksen-Hughes P. (2019). Approaches and methods to measure oxidative stress in clinical samples: research applications in the cancer field. *Oxid. Med. Cell. Longev.* 2019 1–29. 10.1155/2019/1279250 30992736 PMC6434272

[B80] Kehrer J. P. Klotz L.-O. (2015). Free radicals and related reactive species as mediators of tissue injury and disease: implications for Health. *Crit. Rev. Toxicol.* 45 765–798. 10.3109/10408444.2015.1074159 26610815

[B81] Khan S. A. (2020). *Metal Nanoparticles Toxicity: Role of Physicochemical Aspects.* Amsterdam: Elsevier Inc.

[B82] Kim C.-S. Nguyen H.-D. Ignacio R. M. Kim J.-H. Cho H.-C. Maeng E. H. (2014). Immunotoxicity of zinc oxide nanoparticles with different size and electrostatic charge. *Int. J. Nanomedicine* 9 (Suppl. 2), 195–205. 10.2147/IJN.S57935 25565837 PMC4279726

[B83] Kim Y. S. Kim J. S. Cho H. S. Rha D. S. Kim J. M. Park J. D. (2008). Twenty-eight-day oral toxicity, genotoxicity, and gender-related tissue distribution of silver nanoparticles in sprague-dawley rats. *Inhal. Toxicol.* 20 575–583. 10.1080/08958370701874663 18444010

[B84] Klȩbowski B. Depciuch J. Parlińska-Wojtan M. Baran J. (2018). Applications of noble metal-based nanoparticles in medicine. *Int. J. Mol. Sci.* 19:4031. 10.3390/ijms19124031 30551592 PMC6320918

[B85] Kumar R. Roy I. Ohulchanskky T. Y. Vathy L. A. Bergey E. J. Sajjad M. (2010). In vivo biodistribution and clearance studies using multimodal organically modified silica nanoparticles. *ACS Nano* 4 699–708. 10.1021/nn901146y 20088598 PMC2827663

[B86] Kumbhakar D. V. Datta A. K. Mandal A. Das D. Gupta S. Ghosh B. (2016). Effectivity of copper and cadmium sulphide nanoparticles in mitotic and meiotic cells of *Nigella sativa* L. (black cumin) – can nanoparticles act as mutagenic agents? *J. Exp. Nanosci.* 11 823–839. 10.1080/17458080.2016.1149236

[B87] Laha D. Pramanik A. Maity J. Mukherjee A. Pramanik P. Laskar A. (2014). Interplay between autophagy and apoptosis mediated by copper oxide nanoparticles in human breast cancer cells MCF7. *Biochim. Biophys. Acta Gen. Subj.* 1840 1–9. 10.1016/j.bbagen.2013.08.011 23962629

[B88] Lammel T. Mackevica A. Johansson B. R. Sturve J. (2019). Endocytosis, intracellular fate, accumulation, and agglomeration of titanium dioxide (TiO 2) nanoparticles in the rainbow trout liver cell line RTL-W1. *Environ. Sci. Pollut. Res.* 26 15354–15372. 10.1007/s11356-019-04856-1 30929178 PMC6529399

[B89] Lanone S. Rogerieux F. Geys J. Dupont A. Maillot-Marechal E. Boczkowski J. (2009). Comparative toxicity of 24 manufactured nanoparticles in human alveolar epithelial and macrophage cell lines. *Part. Fibre Toxicol.* 6:14. 10.1186/1743-8977-6-14 19405955 PMC2685765

[B90] Lee I. C. Ko J. W. Park S. H. Shin N. R. Shin I. S. Moon C. (2016). Comparative toxicity and biodistribution assessments in rats following subchronic oral exposure to copper nanoparticles and microparticles. *Part. Fibre Toxicol.* 13 1–16. 10.1186/s12989-016-0169-x 27788687 PMC5084351

[B91] Levard C. Hotze E. M. Colman B. P. Dale A. L. Truong L. Yang X. Y. (2013). Sulfidation of silver nanoparticles: natural antidote to their toxicity. *Environ. Sci. Technol.* 47 13440–13448. 10.1021/es403527n 24180218 PMC4019074

[B92] Liu W. Liu S. Wang L. (2019). Surface modification of biomedical titanium alloy: micromorphology. Microstructure evolution and biomedical applications. *Coatings* 9:249 10.3390/coatings9040249

[B93] Lu G. W. Gao P. (2010). “CHAPTER 3 - emulsions and microemulsions for topical and transdermal drug delivery,” in *Handbook of Non-Invasive Drug Delivery Systems*, ed. Kulkarni V. S. (Boston: William Andrew Publishing), 59–94.

[B94] Lu X. Miousse I. R. Pirela S. V. Moore J. K. Melnyk S. Koturbash I. (2016). In vivo epigenetic effects induced by engineered nanomaterials: a case study of copper oxide and laser printer-emitted engineered nanoparticles. *Nanotoxicology* 10 629–639. 10.3109/17435390.2015.1108473 26559097 PMC4958020

[B95] Luzio J. P. Hackmann Y. Dieckmann N. M. G. Griffiths G. M. (2014). The biogenesis of lysosomes and lysosome-related organelles. *Cold Spring Harb. Perspect. Biol.* 6:a016840. 10.1101/cshperspect.a016840 25183830 PMC4142962

[B96] Manshian B. B. Pokhrel S. Mädler L. Soenen S. J. (2018). The impact of nanoparticle-driven lysosomal alkalinization on cellular functionality. *J. Nanobiotechnol.* 16 1–13. 10.1186/s12951-018-0413-7 30382919 PMC6208102

[B97] Marambio-Jones C. Hoek E. M. V. (2010). A review of the antibacterial effects of silver nanomaterials and potential implications for human health and the environment. *J. Nanoparticle Res.* 12 1531–1551. 10.1007/s11051-010-9900-y

[B98] Martirosyan A. Bazes A. Schneider Y. J. (2014). In vitro toxicity assessment of silver nanoparticles in the presence of phenolic compounds-preventive agents against the harmful effect? *Nanotoxicology* 8 573–582. 10.3109/17435390.2013.812258 23738887

[B99] Miyayama T. Matsuoka M. (2016). Involvement of lysosomal dysfunction in silver nanoparticle-induced cellular damage in A549 human lung alveolar epithelial cells. *J. Occup. Med. Toxicol.* 11:1. 10.1186/s12995-016-0090-0 26759602 PMC4709927

[B100] Mohammed Y. H. Holmes A. Haridass I. N. Sanchez W. Y. Studier H. Grice J. E. (2019). Support for the safe use of zinc oxide nanoparticle sunscreens: lack of skin penetration or cellular toxicity after repeated application in volunteers. *J. Invest. Dermatol.* 139 308–315. 10.1016/j.jid.2018.08.024 30448212

[B101] Mordorski B. Friedman A. (2017). “Metal nanoparticles for microbial infection,” in *Functionalized Nanomaterials for the Management of Microbial Infection*, eds R. Boukherroub, S. Szunerits, and D. Drider (Amsterdam: Elsevier), 77–109.

[B102] Namvar F. Rahman H. S. Mohamad R. Azizi S. Tahir P. M. Chartrand M. S. (2015). Cytotoxic effects of biosynthesized zinc oxide nanoparticles on murine cell lines. evidence-based complement. *Altern. Med.* 2015:593014. 10.1155/2015/593014 25784947 PMC4345278

[B103] Nel L. N. Xia A. Mädler L. Li N. (2006). Toxic potential of materials at the nanolevel. *Science* 311 622–627. 10.1126/science.1114397 16456071

[B104] Nemmar A. Hoet P. H. M. Vanquickenborne B. Dinsdale D. Thomeer M. Hoylaerts M. F. (2002). Passage of inhaled particles into the blood circulation. *Hum. Circ.* 106 411–414. 10.1161/01.CIR.0000037134.24080.42 11815420

[B105] Ng C. T. Yong L. Q. Hande M. P. Ong C. N. Yu L. E. Bay B. H. (2017). Zinc oxide nanoparticles exhibit cytotoxicity and genotoxicity through oxidative stress responses in human lung fibroblasts and Drosophila melanogaster. *Int. J. Nanomedicine* 12 1621–1637. 10.2147/IJN.S124403 28280330 PMC5339013

[B106] Osmond-McLeod M. J. Osmond R. I. Oytam Y. McCall M. J. Feltis B. Mackay-Sim A. (2013). Surface coatings of ZnO nanoparticles mitigate differentially a host of transcriptional, protein and signalling responses in primary human olfactory cells. Part. *Fibre Toxicol.* 10:54. 10.1186/1743-8977-10-54 24144420 PMC4016547

[B107] Pacheco I. I. Robbie K. Buzea C. (2007). Nanomaterials and nanoparticles. *Sour. Toxic.* 2 17–71. 10.1116/1.2815690 20419892

[B108] Pan Y. Leifert A. Ruau D. Neuss S. Bornemann J. Schmid G. (2009). Gold nanoparticles of diameter 1.4 nm trigger necrosis by oxidative stress and mitochondrial damage. *Small* 5 2067–2076. 10.1002/smll.200900466 19642089

[B109] Pan Y. Neuss S. Leifert A. Fischler M. Wen F. Simon U. (2007). Size-dependent cytotoxicity of gold nanoparticles. *Small* 3 1941–1949. 10.1002/smll.200700378 17963284

[B110] Park E.-J. Choi J. Park Y.-K. Park K. (2008). Oxidative stress induced by cerium oxide nanoparticles in cultured BEAS-2B cells. *Toxicology* 245 90–100. 10.1016/j.tox.2007.12.022 18243471

[B111] Park H.-J. Kim J. Y. Kim J. Lee J.-H. Hahn J.-S. Gu M. B. (2009). Silver-ion-mediated reactive oxygen species generation affecting bactericidal activity. *Water Res.* 43 1027–1032. 10.1016/j.watres.2008.12.002 19073336

[B112] Pedata P. Ricci G. Malorni L. Venezia A. Cammarota M. Volpe M. G. (2019). In vitro intestinal epithelium responses to titanium dioxide nanoparticles. *Food Res. Int.* 119 634–642. 10.1016/j.foodres.2018.10.041 30884698

[B113] Pelkonen K. H. O. Heinonen-Tanski H. Hänninen O. O. P. (2003). Accumulation of silver from drinking water into cerebellum and musculus soleus in mice. *Toxicology* 186 151–157. 10.1016/S0300-483X(02)00743-6 12604179

[B114] Pizzino G. Irrera N. Cucinotta M. Pallio G. Mannino F. Arcoraci V. (2017). Oxidative stress: harms and benefits for human health. *Oxid. Med. Cell. Longev.* 2017 1–13. 10.1155/2017/8416763 28819546 PMC5551541

[B115] Reidy B. Haase A. Luch A. Dawson K. A. Lynch I. (2013). Mechanisms of silver nanoparticle release, transformation and toxicity: a critical review of current knowledge and recommendations for future studies and applications. *Materials* 6 2295–2350. 10.3390/ma6062295 28809275 PMC5458943

[B116] Roane T. M. Rensing C. Pepper I. L. Maier R. M. (2009). “Chapter 21 - Microorganisms and Metal Pollutants,” in *Environmental Microbiology*, 2nd Edn, eds Pepper I. L. Gerba C. P. Gentry T. J. Maier R. M. (San Diego: Academic Press), 421–441.

[B117] Ryu H.-W. Lee D. H. Florens L. Swanson S. K. Washburn M. P. Kwon S. H. (2014). Analysis of the heterochromatin protein 1 (HP1) interactome in *Drosophila*. *J. Proteomics* 102 137–147. 10.1016/j.jprot.2014.03.016 24681131

[B118] Saliani M. Jalal R. Goharshadi E. K. (2016). Mechanism of oxidative stress involved in the toxicity of ZnO nanoparticles against eukaryotic cells. *Nanomedicine J.* 3 1–14. 10.7508/nmj.2016.01.001

[B119] Samberg M. E. Oldenburg S. J. Monteiro-Riviere N. A. (2010). Evaluation of silver nanoparticle toxicity in skin in vivo and keratinocytes in vitro. *Environ. Health Perspect.* 118 407–413. 10.1289/ehp.0901398 20064793 PMC2854771

[B120] Semmler-Behnke M. Kreyling W. G. Lipka J. Fertsch S. Wenk A. Takenaka S. (2008). Biodistribution of 1.4- and 18-nm gold particles in rats. *Small* 4 2108–2111. 10.1002/smll.200800922 19031432

[B121] Shah J. Bhagat S. Singh S. (2020). “Standard biological assays to estimate nanoparticle toxicity and biodistribution,” in *Nanotoxicity*, eds S. Rajendran, A. Mukherjee, T.A. Nguyen, C. Godugu, and R.K. Shukla (Amsterdam: Elsevier), 71–104.

[B122] Sharma V. Singh P. Pandey A. K. Dhawan A. (2012). Induction of oxidative stress, DNA damage and apoptosis in mouse liver after sub-acute oral exposure to zinc oxide nanoparticles. *Mutat. Res. Genet. Toxicol. Environ. Mutagen.* 745 84–91. 10.1016/j.mrgentox.2011.12.009 22198329

[B123] Shi X. Zhu Y. Hua W. Ji Y. Ha Q. Han X. (2016). An in vivo study of the biodistribution of gold nanoparticles after intervaginal space injection in the tarsal tunnel. *Nano Res.* 9 2097–2109. 10.1007/s12274-016-1100-3

[B124] Shukla R. Bansal V. Chaudhary M. Basu A. Bhonde R. R. Sastry M. (2005). Biocompatibility of gold nanoparticles and their endocytotic fate inside the cellular compartment: a microscopic overview. *Langmuir* 21 10644–10654. 10.1021/la0513712 16262332

[B125] Sintubin L. Verstraete W. Boon N. (2012). Biologically produced nanosilver: current state and future perspectives. *Biotechnol. Bioeng.* 109 2422–2436. 10.1002/bit.24570 22674445

[B126] Soenen S. J. Parak W. J. Rejman J. Manshian B. (2015). (Intra)cellular stability of inorganic nanoparticles: effects on cytotoxicity, particle functionality, and biomedical applications. *Chem. Rev.* 115 2109–2135. 10.1021/cr400714j 25757742

[B127] Sonavane G. Tomoda K. Makino K. (2008). Biodistribution of colloidal gold nanoparticles after intravenous administration: effect of particle size. *Colloids Surf. B Biointerfaces* 66 274–280. 10.1016/j.colsurfb.2008.07.004 18722754

[B128] Stoccoro A. Di Bucchianico S. Coppedè F. Ponti J. Uboldi C. Blosi M. (2017). Multiple endpoints to evaluate pristine and remediated titanium dioxide nanoparticles genotoxicity in lung epithelial A549 cells. *Toxicol. Lett.* 276 48–61. 10.1016/j.toxlet.2017.05.016 28529146

[B129] Sukhanova A. Bozrova S. Sokolov P. Berestovoy M. Karaulov A. Nabiev I. (2018). Dependence of nanoparticle toxicity on their physical and chemical properties. *Nanoscale Res. Lett.* 13:44. 10.1186/s11671-018-2457-x 29417375 PMC5803171

[B130] Suman T. Y. Radhika Rajasree S. R. Kirubagaran R. (2015). Evaluation of zinc oxide nanoparticles toxicity on marine algae chlorella vulgaris through flow cytometric, cytotoxicity and oxidative stress analysis. *Ecotoxicol. Environ. Saf.* 113 23–30. 10.1016/j.ecoenv.2014.11.015 25483368

[B131] Talamini L. Violatto M. B. Cai Q. Monopoli M. P. Kantner K. Krpetiæ Ž, et al. (2017). Influence of size and shape on the anatomical distribution of endotoxin-free gold nanoparticles. *ACS Nano* 11 5519–5529. 10.1021/acsnano.7b00497 28558193

[B132] Thai S. F. Wallace K. A. Jones C. P. Ren H. Prasad R. Y. Ward W. O. (2015). Signaling pathways and microRNA changes in nano-TIO2 treated human lung epithelial (BEAS-2B) cells. *J. Nanosci. Nanotechnol.* 15 492–503. 10.1166/jnn.2015.9202 26328389

[B133] Thevenot P. Cho J. Wavhal D. Timmons R. B. Tang L. (2008). Surface chemistry influences cancer killing effect of TiO2 nanoparticles. *Nanomedicine* 4 226–236. 10.1016/j.nano.2008.04.001 18502186 PMC2597280

[B134] Thit A. Selck H. Bjerregaard H. F. (2015). Toxic mechanisms of copper oxide nanoparticles in epithelial kidney cells. *Toxicol. Vitr.* 29 1053–1059. 10.1016/j.tiv.2015.03.020 25862124

[B135] Tiwari D. K. Jin T. Behari J. (2011). Dose-dependent in-vivo toxicity assessment of silver nanoparticle in Wistar rats. *Toxicol. Mech. Methods* 21 13–24. 10.3109/15376516.2010.529184 21080782

[B136] Trop M. Novak M. Rodl S. Hellbom B. Kroell W. Goessler W. (2006). Silver-coated dressing acticoat caused raised liver enzymes and argyria-like symptoms in burn patient. *J. Trauma Acute Care Surg.* 60:1024. 10.1097/01.ta.0000208126.22089.b6 16531870

[B137] Tsukahara H. (2007). Biomarkers for oxidative stress: clinical application in pediatric medicine. *Curr. Med. Chem.* 14 339–351. 10.2174/092986707779941177 17305536

[B138] U.S. Food and Drug Administration (2019). *USFDA, Code of Federal Regulations.* Silver Spring, FL: Food and drug:, 21.

[B139] Valko M. Morris H. Cronin M. T. D. (2005). Metals, toxicity and oxidative stress. *Curr. Med. Chem* 12 1161–1208. 10.2174/0929867053764635 15892631

[B140] Vigneshwaran N. Varadarajan P. V. Balasubramanya R. H. (2010). “Application of Metallic Nanoparticles in Textiles,” in *Nanotechnologies Life Science*, ed. Ram P. (Weinheim: Wiley-VCH Verlag GmbH & Co. KGaA).

[B141] Vuong N. Q. Goegan P. Mohottalage S. Breznan D. Ariganello M. Williams A. (2016). Proteomic changes in human lung epithelial cells (A549) in response to carbon black and titanium dioxide exposures. *J. Proteomics* 149 53–63. 10.1016/j.jprot.2016.03.046 27084686

[B142] Waghmode M. S. Gunjal A. B. Mulla J. A. Patil N. N. Nawani N. N. (2019). Studies on the titanium dioxide nanoparticles: biosynthesis, applications and remediation. *SN Appl. Sci.* 1:310 10.1007/s42452-019-0337-3

[B143] Wan R. Mo Y. Feng L. Chien S. Tollerud D. J. Zhang Q. (2012). DNA damage caused by metal nanoparticles: the involvement of oxidative stress and activation of ATM Rong. *Chem Res Toxicol.* 25 1402–1411. 10.1021/tx200513t 22559321 PMC3398242

[B144] Wang L. Xie L. Shen P. Fan Q. Wang W. Wang K. (2019). Surface microstructure and mechanical properties of Ti-6Al-4V/Ag nanocomposite prepared by FSP. *Mater. Charact.* 153 175–183. 10.1016/j.matchar.2019.05.002

[B145] Wang W. Han P. Peng P. Zhang T. Liu Q. Yuan S.-N. (2019). Friction stir processing of magnesium alloys: a review. *Acta Metall. Sin. English Lett.* 33 43–57. 10.1007/s40195-019-00971-7

[B146] Wang X. Xia T. Duch M. C. Ji Z. Zhang H. Li R. (2012). Pluronic F108 coating decreases the lung fibrosis potential of multiwall carbon nanotubes by reducing lysosomal injury. *Nano Lett.* 12 3050–3061. 10.1021/nl300895y 22546002 PMC4143198

[B147] Wijnhoven S. W. P. Peijnenburg W. J. G. M. Herberts C. A. Hagens W. I. Oomen A. G. Heugens E. H. W. (2009). Nano-silver – a review of available data and knowledge gaps in human and environmental risk assessment. *Nanotoxicology* 3 109–138. 10.1080/17435390902725914

[B148] Xia Y. Li M. Peng T. Zhang W. Xiong J. Hu Q. (2013). In vitro cytotoxicity of fluorescent silica nanoparticles hybridized with aggregation-induced emission luminogens, for living cell imaging. *Int. J. Mol. Sci.* 14 1080–1092. 10.3390/ijms14011080 23296280 PMC3565308

[B149] Xie Y. Williams N. G. Tolic A. Chrisler W. B. Teeguarden J. G. Maddux B. L. S. (2012). Aerosolized ZnO nanoparticles induce toxicity in alveolar type II epithelial cells at the air-liquid interface. *Toxicol. Sci.* 125 450–461. 10.1093/toxsci/kfr251 21964423 PMC3262851

[B150] Xu F. Piett C. Farkas S. Qazzaz M. Syed N. I. (2013). Silver nanoparticles (AgNPs) cause degeneration of cytoskeleton and disrupt synaptic machinery of cultured cortical neurons. *Mol. Brain* 6:29. 10.1186/1756-6606-6-29 23782671 PMC3695839

[B151] Xu J. Shi H. Ruth M. Yu H. Lazar L. Zou B. (2013). Acute toxicity of intravenously administered titanium dioxide nanoparticles in mice. *PLoS One* 8:e70618. 10.1371/journal.pone.0070618 23950972 PMC3738549

[B152] Xu L. Shi C. Shao A. Li X. Cheng X. Ding R. (2015). Toxic responses in rat embryonic cells to silver nanoparticles and released silver ions as analyzed via gene expression profiles and transmission electron microscopy. *Nanotoxicology* 9 513–522. 10.3109/17435390.2014.948942 25119417

[B153] Yah C. S. (2013). The toxicity of gold nanoparticles in relation to their physiochemical properties. *Biomed. Res.* 24 400–413.

[B154] Yamanaka M. Hara K. Kudo J. (2005). Bactericidal actions of a silver ion solution on *Escherichia coli* Studied by energy-filtering transmission electron microscopy and proteomic analysis. *Appl. Environ. Microbiol.* 71 7589–7593. 10.1128/AEM.71.11.7589-7593.2005 16269810 PMC1287701

[B155] Yang H. Liu C. Yang D. Zhang H. Xi Z. (2009). Comparative study of cytotoxicity, oxidative stress and genotoxicity induced by four typical nanomaterials: the role of particle size, shape and composition. *J. Appl. Toxicol.* 29 69–78. 10.1002/jat.1385 18756589

[B156] Yang W. Shen C. Ji Q. An H. Wang J. Liu Q. (2009). Food storage material silver nanoparticles interfere with DNA replication fidelity and bind with DNA. *Nanotechnology* 20:085102 10.1088/0957-4484/20/8/085102 19417438

[B157] Yang Z. Gu H. Sha G. Lu W. Yu W. Zhang W. (2018). TC4/Ag metal matrix nanocomposites modified by friction stir processing: surface characterization. antibacterial property, and cytotoxicity in vitro. *ACS Appl. Mater. Interfaces* 10 41155–41166. 10.1021/acsami.8b16343 30403843

[B158] Yen H.-J. Hsu S. Tsai C.-L. (2009). Cytotoxicity and immunological response of gold and silver nanoparticles of different sizes. *Small* 5 1553–1561. 10.1002/smll.200900126 19326357

[B159] Yu K. Yoon T. Minai-tehrani A. Kim J. Jin S. Sook M. (2013). Toxicology in Vitro Zinc oxide nanoparticle induced autophagic cell death and mitochondrial damage via reactive oxygen species generation. *Toxicol. Vitr.* 27 1187–1195. 10.1016/j.tiv.2013.02.010 23458966

[B160] Yu L. E. Lanry Yung L.-Y. Ong C.-N. Tan Y.-L. Suresh Balasubramaniam K. Hartono D. (2007). Translocation and effects of gold nanoparticles after inhalation exposure in rats. *Nanotoxicology* 1 235–242. 10.1080/17435390701763108

[B161] Zhang C. Ding Z. Xie L. Zhang L.-C. Wu L. Fu Y. (2017). Electrochemical and in vitro behavior of the nanosized composites of Ti-6Al-4V and TiO2 fabricated by friction stir process. *Appl. Surf. Sci.* 423 331–339. 10.1016/j.apsusc.2017.06.141

[B162] Zhang S. Gao H. Bao G. (2015). Physical principles of nanoparticle cellular endocytosis. *ACS Nano* 9 8655–8671. 10.1021/acsnano.5b03184 26256227 PMC5681865

[B163] Zhang X. Zhang H. Liang X. Zhang J. Tao W. Zhu X. (2016). Iron oxide nanoparticles induce autophagosome accumulation through multiple mechanism: lysosome impairment, mitochondrial damage and ER stress. *Mol. Pharm.* 13 2578–2587. 10.1021/acs.molpharmaceut.6b00405 27287467

[B164] Zhang X.-F. Gurunathan S. Kim J.-H. (2015). Effects of silver nanoparticles on neonatal testis development in mice. *Int. J. Nanomedicine* 10 6243–6256. 10.2147/IJN.S90733 26491295 PMC4599714

[B165] Zhang Y. Ding Z. Zhao G. Zhang T. Xu Q. Cui B. (2018). Transcriptional responses and mechanisms of copper nanoparticle toxicology on zebrafish embryos. *J. Hazard. Mater.* 344 1057–1068. 10.1016/j.jhazmat.2017.11.039 30216965

[B166] Zhao H. Chen L. Zhong G. Huang Y. Zhang X. Chu C. (2019). Titanium dioxide nanoparticles induce mitochondrial dynamic imbalance and damage in HT22 cells. *J. Nanomater.* 2019 1–16. 10.1155/2019/4607531

[B167] Zhao X. Ng S. Heng B. C. Guo J. Ma L. Tan T. T. Y. (2013). Cytotoxicity of hydroxyapatite nanoparticles is shape and cell dependent. *Arch. Toxicol.* 87 1037–1052. 10.1007/s00204-012-0827-1 22415765

[B168] Zhou X. Zhao L. Luo J. Tang H. Xu M. Wang Y. (2019). The toxic effects and mechanisms of nano-cu on the spleen of rats. *Int. J. Mol. Sci.* 20:1469. 10.3390/ijms20061469 30909528 PMC6471436

[B169] Zhu Y. Eaton J. W. Li C. (2012). Titanium dioxide (TiO2) nanoparticles preferentially induce cell death in transformed cells in a Bak/Bax-independent fashion. *PLoS One* 7:e50607. 10.1371/journal.pone.0050607 23185639 PMC3503962

[B170] Zoroddu M. A. Medici S. Ledda A. Nurchi V. M. Lachowicz J. I. Peana M. (2014). Toxicity of nanoparticles. *Curr. Med. Chem.* 21 3837–3853. 10.2174/0929867321666140601162314 25306903

